# High EBV-reactive IgG levels in serum are linked to typical inflammatory cerebrospinal fluid features in multiple sclerosis

**DOI:** 10.3389/fimmu.2026.1929166

**Published:** 2026-10-05

**Authors:** Stephan Neidhart, Klara Asplund Högelin, Marc Hilty, Benjamin Vlad, Mario Ziegler, Mohsen Khademi, Andreas Lutterotti, Roland Martin, Faiez Al Nimer, Ilijas Jelcic

**Affiliations:** 1 Neuroimmunology and Multiple Sclerosis Research Section, Department of Neurology, University Hospital Zurich and University of Zurich, Zurich, Switzerland; 2 Department of Neurology, Hirslanden Klinik Zurich, Zurich, Switzerland; 3 Center for Multiple Sclerosis and Clinical Neuroimmunology, Bellevue Medical Group, Zurich, Switzerland; 4 Neuroimmunology Unit, Center for Molecular Medicine, Department of Clinical Neuroscience, Karolinska Institutet, Stockholm, Sweden; 5 Department of Laboratory Medicine, Karolinska Institutet, Stockholm, Sweden; 6 Section Translational Neuroimmunology, Department of Neurology, Jena University Hospital, Jena, Germany; 7 Cellerys AG, Schlieren, Switzerland; 8 Institute of Experimental Immunology, University of Zurich, Zurich, Switzerland; 9 Therapeutic Immune Design Unit, Center for Molecular Medicine, Department of Clinical Neuroscience, Karolinska Institutet, Stockholm, Sweden

**Keywords:** cerebrospinal fluid, CSF cell count, EBV-specific immunity, Epstein-Barr virus, intrathecal antibody production, multiple sclerosis, pleocytosis

## Abstract

**Background:**

Epstein-Barr virus (EBV) infection is strongly associated with multiple sclerosis (MS). However, it is ill-defined which of the MS-typical inflammatory alterations of basic cerebrospinal fluid (CSF) parameters, i.e. intrathecal cellular inflammation versus intrathecal total production of antibodies, are primarily linked to EBV-mediated humoral immune signatures in serum and/or CSF.

**Methods:**

We analyzed paired serum and cerebrospinal fluid (CSF) samples from 316 MS patients from Switzerland and Sweden. Serum IgG levels (sIgG) against EBV nuclear antigen 1 (EBNA1) and EBV capsid antigen (EBV-CA) were quantified by ELISA, and high levels (IgG^hi^) were defined as the highest quartile (Q4) of serum EBNA1- or EBV-CA-specific IgG levels (sEBNA1-IgG or sEBV-CA-IgG), respectively. Intrathecal EBV-specific antibody production was defined as CSF/serum antigen-specific antibody index (CAI) ≥1.5. We assessed associations of EBV-specific measures with CSF leukocyte count, presence of pleocytosis, presence of blood-CSF barrier dysfunction, presence of CSF-specific oligoclonal bands (OCB) and intrathecal immunoglobulin indices/fractions using correction for multiple comparisons, as well as age- and sex-adjusted models and continuous dose–response analyses.

**Results:**

sEBNA1-IgG^hi^ levels were associated with higher CSF leukocyte counts (median 6.0 vs 3.0 cells/µL; q=0.022), more frequent pleocytosis (55.7% vs 37.6%; q=0.022), and higher rate of CSF-specific OCB (93.7% vs 79.3%; q=0.029) compared to lower sEBNA1-IgG levels. sEBV-CA-IgG^hi^ levels were not associated with cellular CSF parameters, but with a higher total IgG index (0.78 vs 0.65; q=0.016). In age- and sex-adjusted models, a 10-fold increase in sEBNA1-IgG levels was associated with a 2.54-fold higher CSF leukocyte count (β=0.405, 95% CI 0.217–0.592, p=2.86×10^–5^) and 2.73-fold higher odds for pleocytosis (OR = 2.73, 95% CI 1.59–4.71, p=2.83×10^–4^), whereas sEBV-CA-IgG showed no significant correlation. Dose-response models showed monotonic associations between sEBNA1-IgG levels and both CSF leukocyte counts and general intrathecal humoral activation, whereas sEBV-CA-IgG levels were associated mainly with overall intrathecal humoral activity. Intrathecal EBV-specific antibody production was uncommon and not associated with basic CSF inflammatory or barrier parameters.

**Conclusion:**

These findings are consistent with an outside-in model, in which peripheral, but not intrathecal activation of EBV-specific humoral immunity, and rather sEBNA1-IgG^hi^- than sEBV-CA-IgG^hi^-reactivity links to general intrathecal cellular and humoral immune activation in MS.

## 1 Introduction

Multiple sclerosis (MS) is a chronic autoimmune disease of the central nervous system (CNS) characterized by inflammatory cell infiltration and -activation, demyelination, and neuroaxonal injury. Convergent evidence from epidemiology, immunology, and genetics implicates Epstein-Barr virus (EBV) infection as a necessary precursor and key driver for its development, albeit the mechanistic link between EBV and MS is incompletely understood (1–3). Several ways about how dysregulated immune activity against EBV in MS can manifest have been described and discussed: as a systemic immune response (4–6), as compartmentalized locally confined EBV-specific immune responses targeting the CNS (7, 8), or as broader intrathecal humoral immune activation that is not EBV antigen-specific, but associated with EBV-specific seroreactivity (9, 10). Exploring a possible association between EBV immunobiology and the MS-typical intrathecal inflammatory changes would help to elucidate whether and how EBV-related immunity connects to MS pathogenesis.

Routine clinical analysis of cerebrospinal fluid (CSF) provides a direct readout of inflammatory activity within the CNS compartment and captures distinct but complementary dimensions of MS immunopathology. CSF leukocyte count and pleocytosis reflect cellular inflammatory trafficking into the CNS (11), whereas the CSF/serum albumin quotient (Q_Alb_) within the Reiber framework reflects blood-CSF barrier function and CSF flow-related exchange of albumin across this barrier (12). Intrathecal antibody synthesis is a hallmark of MS and is typically captured by the detection of CSF-restricted oligoclonal IgG bands (OCB) or pathological immunoglobulin- or free light chain indices, but also quantitative measures such as intrathecal fractions (IF) of antibodies that estimate the fraction of total CSF immunoglobulins attributable to intrathecal synthesis (11–13). The MRZ reaction, a polyspecific intrathecal antiviral antibody response, constitutes an additional signature of intrathecal B cell activation and is highly specific for MS compared with other inflammatory neurological disorders (14). These MS-typical changes of CSF measures have been known for many decades (11), but are mechanistically not well understood. The study of immune reactivities against specific antigens of the key environmental risk factor, EBV, in the context of these basic CSF measures, which reflect (i) cellular CNS inflammation, (ii) barrier dysfunction/CSF exchange, and (iii) intrathecal B-cell activation, may lead to a better understanding.

EBV-directed immunity can be assessed at multiple levels that likely represent distinct biological processes. Serum IgG against EBV nuclear antigen 1 (EBNA1) and EBV capsid antigen (EBV-CA) reflect the systemic magnitude of EBV-specific humoral immune memory and may be shaped by host genetics, timing of primary EBV infection, development of symptoms during primary EBV infection in the form of infectious mononucleosis, and viral reactivation dynamics (15). In contrast, antigen-specific CSF/serum antibody indices (CAI) attempt to quantify antigen-specific intrathecal antibody production beyond what would be expected from passive transfer across the blood-CSF barrier, thereby providing a proxy for compartmentalized EBV-directed humoral responses within the CNS (16). Prior studies have reported heterogeneous associations between EBV serology and CSF phenotypes in MS, partly explained by differences in cohort composition, assay platforms, outcome definitions, and limited control for multiple comparisons (16–18). Moreover, it remains uncertain whether EBV-specific immune reactions in MS, if present, are rather associated with cellular inflammation or intrathecal humoral activation, or both.

In this study, we explored if higher EBV-specific systemic seroreactivity and/or intrathecal EBV-specific IgG production are associated with (i) increased CSF leukocyte count, (ii) CSF-blood barrier dysfunction and/or (iii) markers of general intrathecal humoral activation.

## 2 Material and methods

### 2.1 Study design and participants

This cross-sectional study included 316 patients with relapsing-remitting MS (RRMS), secondary progressive MS (SPMS) or primary progressive MS (PPMS) from two centers (University Hospital Zurich, n=141; and Karolinska University Hospital Stockholm, n=175) (**Table 1**). All patients fulfilled the 2024 revised McDonald criteria for the diagnosis of MS (19). The study procedures were approved by the Cantonal Ethics Committee of Zurich and the Swedish Ethical Review Authority (EC-No. 2013–0001 and 2022-03650-02). Written informed consent was obtained from all patients or their relatives. Basic demographic, serum, and CSF data were retrospectively collected from electronic records and anonymized. Patients from Zurich with lumbar puncture between 2009–2021 and patients from Stockholm with lumbar puncture between 2009–2017 were included, provided routine CSF parameters, i.e. CSF leukocyte count, CSF/serum albumin quotient (Q_Alb_), total and antiviral IgG levels and OCB from CSF and serum, and paired serum and CSF samples were available for analysis of EBV-specific antibodies. No other inclusion or exclusion criteria were taken into account.

**Table 1:** Demographics of the cohort of MS patients (N = 316).

Demographics and clinical characteristics	Value
- Female sex, n/N (%)	213/316 (67.4%)
- Age at sampling, years, median (IQR)	36.5 (29.0–45.0)
- Age at disease onset, years, median (IQR)	32.0 (25.0–39.2)
- Disease duration, months, median (IQR)	10.7 (1.4–90.1)
- Therapy-naïve* patients, n/N (%)	278/316 (88.0%)
Disease course	Value
- Relapsing-remitting MS (RRMS), n/N (%)	274/316 (86.7%)
- Primary progressive MS (PPMS), n/N (%)	19/316 (6.0%)
- Secondary progressive MS (SPMS), n/N (%)	23/316 (7.3%)

Data are median (interquartile range, IQR) or n/N (%).

*Therapy-naïve is defined as untreated patients or patients who have previously been treated with any of the further listed medications and have been off therapy for the respective time-periods: (i) corticosteroids within 4 weeks before LP, (ii) glatiramer acetate, interferon-beta and dimethylfumarate within 2 months before LP, (iii) teriflunomide, fingolimod and natalizumab within 3 months before LP, (iv) ocrelizumab, rituximab, mitoxantrone within 12 months before LP, (v) aHSCT within 2 years before LP.

### 2.2 Laboratory measurements and definitions

All basic serum and CSF parameters retrieved from electronic patient charts had been measured in the clinical laboratories of the two centers mentioned above as part of routine clinical analysis. CSF cellular inflammatory activity is routinely assessed by CSF leukocyte count (cells/µL) and presence of pleocytosis, defined as >4 cells/µL, following standardized protocols as previously described (20). Levels of albumin, total IgG, total IgA, and total IgM in serum and CSF are quantified by immunonephelometry, and OCB are detected by isoelectric focusing on agarose gels and immunoblot with IgG-specific antibodies in a semi-automated assay as described before (21). Blood-CSF barrier function is evaluated using the CSF/serum albumin quotient (Q_Alb_, ×10^-3^), and blood-CSF barrier dysfunction is defined according to Reiber’s age-adjusted Q_Alb_ reference limit (12). Intrathecal humoral immune activation is assessed using CSF-restricted oligoclonal bands (OCB; type 2 or 3) (22), immunoglobulin indices (IgG-, IgM- and IgA indices), and measures of intrathecal immunoglobulin synthesis, including intrathecal fraction (IF), according to Reiber (12), where available. For IF analyses, IF>0% was interpreted as evidence of intrathecal synthesis (12), and analysis of IF intensity (%) was restricted to patients with IF>0%. **Table 2** summarizes the basic CSF parameter findings.

**Table 2:** Basic CSF characteristics of the cohort of MS patients (n=316).

CSF cell counts and blood-CSF barrier	Value
- CSF leukocyte count, cells/µL, median (IQR)	4.0 (2.0–8.0)
- Pleocytosis (>4 cells/µL), n/N (%)	133/316 (42.1%)
- CSF albumin, mg/L, median (IQR)	209.5 (162.8–280.5)
- CSF/serum albumin quotient (Q_Alb_, ×10^-3^), median (IQR)	4.52 (3.60–6.20)
- Blood-CSF barrier dysfunction, n/N (%)	84/316 (26.6%)
Intrathecal immunoglobulin synthesis	Value
- CSF total IgG, mg/L, median (IQR)	38.4 (26.0–54.5)
- Serum total IgG, g/L, median (IQR)	10.6 (9.3–12.0)
- IgG index, median (IQR)	0.66 (0.53–0.94)
- Elevated IgG index (>0.7), n/N (%)	141/316 (44.6%)
- Intrathecal synthesis of total IgG (Reiber’s IF_IgG_>0%), n/N (%)	153/316 (48.4%)
- Intrathecal synthesis of total IgM (Reiber’s IF_IgM_ >0%), n/N (%)	33/141 (23.4%)
- Intrathecal synthesis of total IgA (Reiber’s IF_IgA_ >0%), n/N (%)	15/141 (10.6%)
- CSF-specific oligoclonal bands (OCB type 2 or 3), n/N (%)	262/316 (82.9%)

Data are median (IQR) or n/N (%). Pleocytosis was defined as >4 cells/µL. Q_Alb_ is unitless and displayed as ×10⁻³. Antibody indices are unitless.

After identifying paired CSF and serum samples, we measured serum and CSF levels of IgG reactive against EBNA1 and EBV-CA using commercial ELISA kits (EI 2793–9601 G, EI 2793-9601-L G, EI 2791–9601 G, EI 2791-9601-L G, Euroimmun AG, Kriens, Switzerland) and fully automated ELISA processing (Analyzer I, Euroimmun AG, Kriens, Switzerland). The anti-EBNA1 IgG assays (EI 2793–9601 G, EI 2793-9601-L G) use recombinant EBNA1 protein, whereas the anti-EBV-CA assays (EI 2791–9601 G, EI 2791-9601-L G) use purified EBV capsid antigens derived from an inactivated lysate of EBV-infected human B cells. To quantify levels of serum IgG reactive against EBNA1 and EBV-CA, serum samples were diluted 1:101, and processed using manufacturer-specified incubation steps of 30, 30, and 15 minutes at room temperature. We modified the standard curve, following discussion with the manufacturer, to a seven-point calibration curve ranging from 0 to 200 relative units per milliliter (RU/mL), thereby enabling quantitative rather than semi-quantitative determination of EBV-specific serum antibody levels. Serum EBV-specific IgG levels were reported as RU/mL.

For CSF/serum antibody index (CAI) measurements with paired CSF/serum testing, standardized dilutions of 1:2 and 1:10 for CSF and 1:404 and 1:2020 for serum were used. Because total IgG and antigen-specific IgG levels are generally much lower in CSF than in serum, lower serum dilutions in the paired assay could result in disproportionately high signals or values exceeding the calibration range. Therefore, serum samples were tested at higher dilutions (1:404 and 1:2020) in the paired CSF/serum assay than in the serum antibody-quantification assay (1:101). For paired CSF/serum testing, a four-point calibration curve was used with calibrators at 5, 25, 50, and 100 RU. If a patient sample yielded an absorbance value outside the calibration range, the serum and/or CSF sample was retested at an appropriate dilution (higher for samples exceeding the range and lower for samples falling below it), until the result fell within the calibration range. The EBNA1- or EBV-CA-specific CSF/serum antibody index (EBNA1-CAI or EBV-CA-CAI, i.e. CAI_spec_) was calculated according to Reiber (12): CAI_spec_ was calculated as CAI_spec_ = Q_spec_/Q_IgG_ (if Q_Lim_ > Q_IgG_), or as CAI_spec_ = Q_spec_/Q_Lim_, (if Q_Lim_ < Q_IgG_); with Q_spec_ = antigen-specific IgG_CSF_ [RU]/antigen-specific IgG_serum_ [RU]; Q_IgG_ = total IgG_CSF_ [mg/l]/total IgG_serum_ [mg/l]; Q_Lim_ = 0.93 × (Q_Alb_^2^ + 6 × 10^−6^)^0.5^ – 1.7 × 10^−3^; Q_Lim_ refers to the upper discrimination line of the hyperbolic reference range for the blood-derived IgG in CSF indicating absence of intrathecal IgG production. Intrathecal EBV-specific antibody production was defined as EBNA1- or EBV-CA-specific CAI≥1.5, which is the conventional cutoff used in Reiber-based analyses. All samples were anonymized after EBV-related measurements and collection of basic serum and CSF parameters and demographic parameters from the patient charts.

### 2.3 Exposure definitions

High serum EBV-specific IgG reactivity was defined as the highest fourth quartile (Q4) of antibody concentrations in the cohort: Q4 serum EBNA1-specific IgG levels (sEBNA1-IgG^hi^) were >336 RU/mL and Q4 serum EBV-CA-specific IgG levels (sEBV-CA-IgG^hi^) were >372 RU/mL. Primary comparisons evaluated Q4 levels versus <Q4 levels for each exposure.

### 2.4 Statistical analysis

Continuous variables are reported as median with interquartile range (IQR) and categorical variables as n/N (%). Group comparisons used Mann–Whitney U tests for continuous outcomes and χ² tests (or Fisher’s exact tests when appropriate) for categorical outcomes. To assess relationships between variables, we conducted Pearson and Spearman correlation analyses by calculating Pearson’s correlation coefficient (r) and Spearman’s rank-order correlation (ρ), and two-tailed p<0.05 was considered statistically significant. Both correlation coefficients were calculated for all variable pairs to provide comprehensive assessment: Pearson identified linear relationships in normally distributed data, while Spearman captured relationships that violated parametric assumptions or exhibited monotonic but non-linear patterns. Discrepancies between coefficients informed interpretation of relationship linearity and outlier sensitivity: When r < ρ, then the relationship is monotonic but non-linear (e.g. logarithmic), or outliers are inflating Spearman’s rank-based measure. When r ≈ ρ, then the relationship is likely linear and data meet parametric assumptions. When both ≈ 0, then no linear relationship exists, although other non-monotonic relationships may still be present.

To address multiplicity, p-values were adjusted using the Benjamini–Hochberg false discovery rate (FDR) (23) within pre-defined families: (i) all outcomes within each stratum (all, female, male) in the comparisons of MS patients with Q4 versus <Q4 levels of serum EBNA1-specific IgG (sEBNA1-IgG^hi^ versus sEBNA1-IgG^lo^) and in the comparisons of MS patients with Q4 versus <Q4 levels of serum EBV-CA-specific IgG (sEBV-CA-IgG^hi^ versus sEBV-CA-IgG^lo^); (ii) outcomes among MS patients with versus without intrathecal EBNA1-specific IgG production (EBNA1-CAI ≥1.5 versus EBNA1-CAI <1.5) and among MS patients with versus without intrathecal EBV-CA-specific IgG production (EBV-CA-CAI ≥1.5 versus EBV-CA-CAI <1.5); and (iii) EBNA1- and EBV-CA-specific serum and intrathecal IgG responses among patients with or without intrathecal production of total IgG, total IgA or total IgM. Both unadjusted p-values and FDR q-values are reported.

Minimal adjusted models assessed associations between serum EBV-specific IgG levels and basic parameters of CSF inflammation, adjusting for age at sampling and sex. Linear models used log1p (CSF leukocyte count), and logistic models used pleocytosis as outcome; exposures were expressed per 10-fold increase in antibody concentration (log10 RU/mL). Robust (Huber) regression was used as sensitivity analysis for linear models (24).

## 3 Results

### 3.1 Cohort characteristics

A total of 316 MS patients were included in the study (**Table 1**). Most patients had RRMS (86.7%), while PPMS and SPMS accounted for 6.0% and 7.3%, respectively. Pleocytosis (>4 cells/µL) was present in 42.1% (**Table 2**). Blood-CSF barrier dysfunction was present in 26.6%. CSF-restricted OCB (OCB type 2 or 3) were observed in 82.9%. 25.0% and 24.7% were classified as Q4 for sEBNA1-IgG and sEBV-CA-IgG, respectively (**Table 3**). Intrathecal EBV-specific antibody production (CAI ≥1.5) was uncommon (EBNA1-CAI ≥1.5: 5.8%; EBV-CA-CAI ≥1.5: 7.1%). The distributions of EBNA1- and EBV-CA-specific IgG levels in serum and in CSF, as well as the antigen-specific CSF/serum antibody indices (EBNA1-CAI and EBV-CA-CAI values) are shown in **Supplementary Figure 1**.

**Table 3:** EBV-specific IgG reactivities in serum and CSF of the cohort of MS patients (N = 316).

Serum Epstein-Barr virus-specific antibody levels	Value
- Serum EBNA1-specific IgG (sEBNA1-IgG), RU/mL, median (IQR)	173.1 (117.9–346.7)
- Serum EBV-CA-specific IgG (sEBV-CA-IgG), RU/mL, median (IQR)	183.6 (136.4–366.5)
- sEBNA1-IgG^hi^ (Q4, >336 RU/mL), n/N (%)	79/316 (25.0%)
- sEBV-CA-IgG^hi^ (Q4, >372 RU/mL), n/N (%)	78/316 (24.7%)
Intrathecal Epstein-Barr virus-specific antibody production	Value
- EBNA1-specific CSF/serum antibody index (EBNA1-CAI), median (IQR)	0.5 (0.4–0.7)
- EBV-CA-specific CSF/serum antibody index (EBV-CA-CAI), median (IQR)	0.6 (0.5–0.8)
- EBNA1-specific CSF/serum antibody index (EBNA1-CAI) ≥1.5, n/N (%)	18/309 (5.8%)
- EBV-CA-specific CSF/serum antibody index (EBV-CA-CAI) ≥1.5, n/N (%)	22/309 (7.1%)

Data are median (IQR) or n/N (%). Antibody indices are unitless.

### 3.2 Correlations between serum EBV-specific IgG levels and CSF leukocyte count

In the overall cohort, sEBNA1-IgG levels correlated positively with CSF leukocyte count (Pearson r=0.155, p=0.0057; Spearman ρ=0.221, p=7.5×10^–5^) (**Figure 1**). In contrast, sEBV-CA-IgG levels did not correlate with CSF leukocyte count (r=0.010, p=0.86; ρ=0.061, p=0.28). In sex-stratified analyses, the association between sEBNA1-IgG levels and CSF leukocyte count was more evident in females, whereas estimates in males were less consistent (**Supplementary Figure 2**). There were no significant sex-specific associations between sEBV-CA-IgG levels and CSF leukocyte counts (**Supplementary Figure 2**).

**Figure 1:**
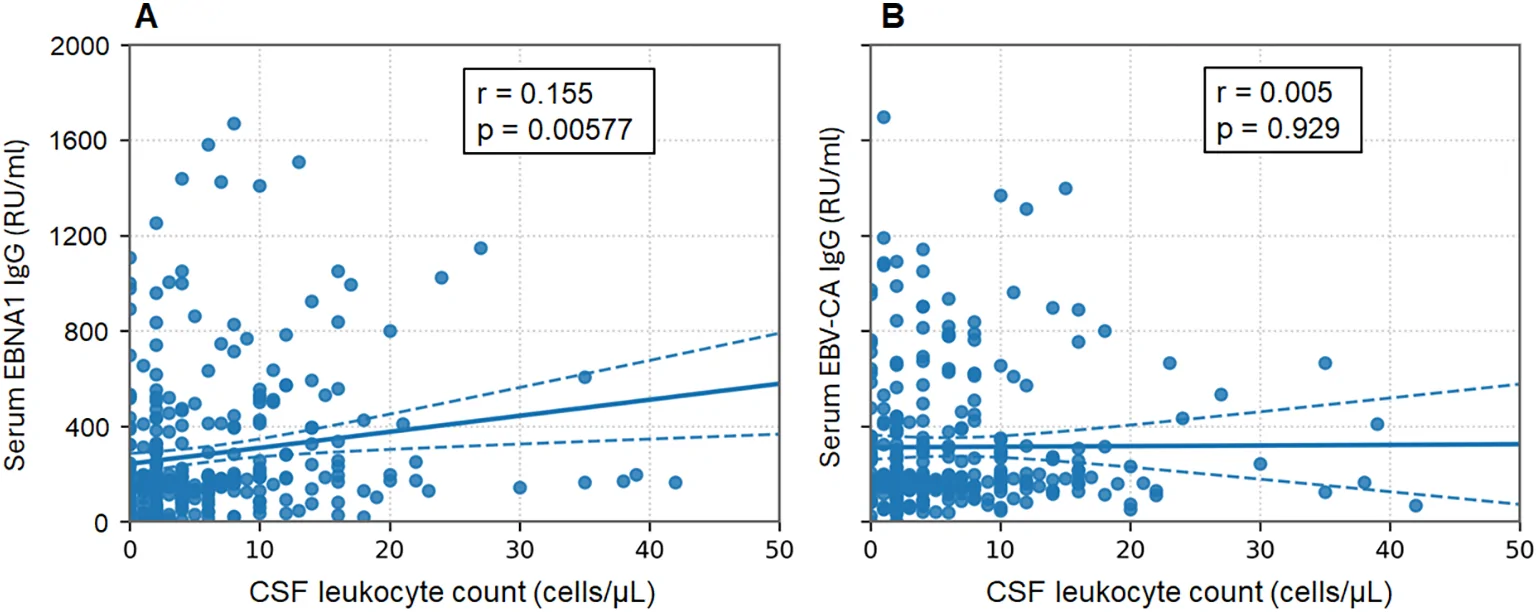
Association between serum EBNA1-specific IgG levels and CSF leukocyte count. Scatter plot of serum EBNA1-specific IgG levels (RU/mL; y-axis) versus CSF leukocyte count (cells/µL; x-axis) in the overall cohort. The solid line indicates the fitted linear regression, with the shaded band showing the 95% confidence interval. The correlation coefficient (Pearson’s r) and corresponding p value are displayed in the panel. Each dot represents one the values of a single patient.

### 3.3 Associations between high EBV-specific IgG levels and general markers of CSF inflammation

MS patients with high, i.e. Q4, sEBNA1-IgG levels (sEBNA1-IgG^hi^) showed higher CSF leukocyte counts compared with MS patients with lower, i.e. <Q4, sEBNA1-IgG levels (sEBNA1-IgG^lo^) (p=0.0052; FDR q=0.0223) and a higher frequency of pleocytosis (p=0.0047; q=0.0223) (**Table 4**). sEBNA1-IgG^hi^ levels were also associated with higher rates of CSF-specific OCB (p=0.0033; q=0.0223). Q_Alb_ and blood-CSF barrier dysfunction did not differ after FDR correction. In females, sEBNA1-IgG^hi^ levels associated with higher CSF leukocyte counts (p=0.0043; q=0.0278) as well as higher rates of CSF-specific OCB (p=0.0014; q=0.0182) (**Supplementary Table 1**), whereas male-stratified comparisons did not retain FDR significance (**Supplementary Table 2**).

**Table 4:** Differences in basic CSF parameters among MS patients with and without high serum EBNA1-specific IgG levels (Q4 versus <Q4). High serum EBNA1-specific IgG levels (sEBNA1-IgG^hi^) were defined as the highest quartile (Q4) of antibody concentrations in the cohort, i.e. sEBNA1-IgG >336 RU/mL, and low serum EBNA1-specific IgG levels (sEBNA1-IgG^lo^) were defined as lower three quartiles (<Q4) of antibody concentrations in the cohort, i.e. sEBNA1-IgG <336 RU/mL.

Outcome	sEBNA1-IgG^hi^ (Q4)	sEBNA1-IgG^lo^ (<Q4)	p-value	FDR q-value
n/N (%)	79/316 (25.0%)	237/316 (75.0%)	–	–
CSF leukocyte count, cells/µL, median (IQR)	6.0 (2.0–11.0)	3.0 (2.0–7.0)	0.0052	**0.0223**
Patients with pleocytosis, n/N (%)	44/79 (55.7%)	89/237 (37.6%)	0.0047	**0.0223**
Q_Alb_, median (IQR)	4.99 (3.89–6.68)	4.49 (3.56–6.13)	0.0780	0.1448
BCSFB dysfunction, n/N (%)	22/79 (27.8%)	62/237 (26.2%)	0.7687	0.7724
CSF-specific OCB, n/N (%)	74/79 (93.7%)	188/237 (79.3%)	0.0033	**0.0223**
IgG index, median (IQR)	0.73 (0.59–1.02)	0.65 (0.52–0.92)	0.0264	0.0688
Elevated IgG index (>0.7), n/N (%)	42/79 (53.2%)	99/237 (41.8%)	0.0777	0.1448
Intrathecal synthesis of total IgG (Reiber’s IF_IgG_ >0%), n/N (%)	47/79 (59.5%)	106/237 (44.7%)	0.0229	0.0688
Intensity of IF_IgG_ (%), median (IQR), among patients with IF_IgG_>0% (n)*	32.5 (16.5–52.2) (n=48)	31.8 (14.9–49.5) (n=110)	0.7724	0.7724
Intrathecal synthesis of total IgM (Reiber’s IF_IgM_ >0%), n/N (%)	9/27 (33.3%)	24/114 (21.1%)	0.1754	0.2850
Intensity of IF_IgM_ (%), median (IQR), among patients with IF_IgM_>0% (n)*	46.7 (25.2–58.0) (n=9)	53.7 (28.1–66.8) (n=24)	0.5578	0.6592
Intrathecal synthesis of total IgA (Reiber’s IF_IgA_), n/N (%)	4/27 (14.8%)	11/114 (9.6%)	0.4874	0.6362
Intensity of IF_IgA_ (%), median (IQR), among patients with IF_IgA_>0% (n)*	46.7 (17.6–76.5) (n=4)	25.3 (12.2–35.1) (n=11)	0.4894	0.6362

Continuous variables are reported as median (IQR) (with outcome-specific n); categorical variables as % (with n). P-values are from Mann–Whitney U tests or chi-square/Fisher’s exact tests as appropriate. p-values across outcomes were adjusted using Benjamini–Hochberg; FDR q-values are shown. Intrathecal fraction (IF) summaries are restricted to patients with IF >0%.

BCSFB, blood-CSF barrier; CSF, cerebrospinal fluid; IF, intrathecal fraction; OCB, oligoclonal bands; Q_Alb_, CSF/serum albumin quotient. *n, number of patients with IF_IgG/IgM/IgA_>0%.

Significant findings are marked in bold.

In contrast, sEBV-CA-IgG^hi^ levels showed no association with CSF leukocyte count, pleocytosis, Q_Alb_, blood-CSF barrier dysfunction, or rates of CSF-restricted OCB after FDR correction (**Table 5**). The strongest sEBV-CA-IgG^hi^-associated signals were a higher IgG index (p=0.0024; FDR q=0.0158) and a higher frequency of an elevated (>0.7) IgG index (p=0.0005; FDR q=0.0069). Other outcomes showed only nominal differences that did not remain significant after FDR correction, and no FDR-significant associations were observed in sex-stratified sEBV-CA-IgG analyses (**Supplementary Table 3**, **4**).

**Table 5:** Differences in basic CSF parameters among MS patients with and without very high serum EBV-CA-specific IgG levels (Q4 versus <Q4).

Outcome	sEBV-CA-IgG^hi^ (Q4)	sEBV-CA-IgG^lo^ (<Q4)	p-value	FDR q-value
n/N (%)	78/316 (24.7%)	238/316 (75.3%)	–	–
CSF leukocyte count, cells/µL, median (IQR)	4.0 (2.0–8.0)	4.0 (2.0–9.0)	0.5207	0.6769
Patients with pleocytosis, n/N (%)	36/78 (46.2%)	97/238 (40.8%)	0.4021	0.6769
Q_Alb_, median (IQR)	4.50 (3.60–6.00)	4.61 (3.61–6.22)	0.5071	0.6769
BCSFB dysfunction, n/N (%)	18/78 (23.1%)	66/238 (27.7%)	0.4194	0.6769
CSF-specific OCB, n/N (%)	64/78 (82.1%)	198/238 (83.2%)	0.8161	0.8161
IgG index, median (IQR)	0.78 (0.60–1.12)	0.65 (0.52–0.84)	0.0024	**0.0158**
Elevated IgG index (>0.7), n/N (%)	48/78 (61.5%)	93/238 (39.1%)	0.0005	**0.0069**
Intrathecal synthesis of total IgG (Reiber’s IF_IgG_ >0%), n/N (%)	46/78 (59.0%)	107/238 (45.0%)	0.0316	0.1368
Intensity of IF_IgG_ (%), median (IQR), among patients with IF_IgG_>0% (n)*	39.4 (24.1–51.0) (n=48)	28.9 (13.9–48.7) (n=110)	0.1460	0.3797
Intrathecal synthesis of total IgM (Reiber’s IF_IgM_ >0%), n/N (%)	9/34 (26.5%)	24/107 (22.4%)	0.6278	0.7420
Intensity of IF_IgM_ (%), median (IQR), among patients with IF_IgM_>0% (n)*	28.0 (17.4–53.1) (n=9)	55.1 (29.1–70.2) (n=24)	0.0787	0.2557
Intrathecal synthesis of total IgA (Reiber’s IF_IgA_), n/N (%)	4/34 (11.8%)	11/107 (10.3%)	0.7576	0.8161
Intensity of IF_IgA_ (%), median (IQR), among patients with IF_IgA_>0% (n)*	48.9 (20.8–76.5) (n=4)	25.3 (12.2–35.1) (n=11)	0.4117	0.6769

Continuous variables are reported as median (IQR) (with outcome-specific n); categorical variables as % (with n). P-values are from Mann–Whitney U tests or chi-square/Fisher’s exact tests as appropriate. p-values across outcomes were adjusted using Benjamini–Hochberg; FDR q-values are shown. Intrathecal fraction (IF) summaries are restricted to participants with IF >0.

BCSFB, blood-CSF barrier; CSF, cerebrospinal fluid; IF, intrathecal fraction; OCB, oligoclonal bands; Q_Alb_, CSF/serum albumin quotient. *n, number of patients with IF_IgG/IgM/IgA_>0%.

High serum EBV-CA-specific IgG levels (sEBV-CA-IgG^hi^) were defined as the highest quartile (Q4) of antibody concentrations in the cohort, i.e. sEBV-CA-IgG >372 RU/mL, and low serum EBV-CA-specific IgG levels (sEBV-CA-IgG^lo^) were defined as lower three quartiles (<Q4) of antibody concentrations in the cohort, i.e. sEBV-CA-IgG <372 RU/mL. Significant findings are marked in bold.

In age- and sex-adjusted models, higher sEBNA1-IgG levels were robustly associated with inflammatory CSF outcomes: per 10-fold increase in sEBNA1-IgG levels, CSF leukocyte count (log1p) increased 2.54-fold (β=0.405, 95% CI 0.217–0.592; p=2.86×10^–5^) and the odds of pleocytosis increased 2.73-fold (OR = 2.73, 95% CI 1.59–4.71; p=2.83×10^–4^) (**Supplementary Table 5**). Associations for sEBV-CA-IgG were not significant (cell count β=0.227; p=0.070; pleocytosis OR = 1.92; p=0.050).

To better compare the magnitude of the differences in various basic CSF markers between two groups, rather than just assessing whether a significant difference exists, we calculated group differences using Hedges’ g-standardized effect sizes with 95% confidence intervals (CI) and FDR-adjusted q-values (**Figure 2**). Here, we evaluated the relative magnitudes of differences between standard CSF markers of intrathecal cellular and humoral immune activation across two groups for each EBV antigen-specific serum IgG reactivity: (i) sEBNA1-IgG^hi^ versus sEBNA1-IgG^lo^ levels, and (ii) sEBV-CA-IgG^hi^ versus sEBV-CA-IgG^lo^ levels. sEBNA1-IgG^hi^ levels were consistently associated with elevated key basic parameters of cellular and humoral CSF inflammation, whereas sEBV-CA-IgG^hi^ levels showed smaller, less consistent effects. Sex-stratified analyses revealed that sEBNA1-IgG^hi^-related associations were more pronounced and with a smaller CI in females than in males (**Supplementary Figure 3**). In males, the associations were generally less clear and varied more, which likely reflects the smaller subgroup size. sEBV-CA-IgG^hi^-related effects remained modest in both sexes (**Supplementary Figure 3**).

**Figure 2:**
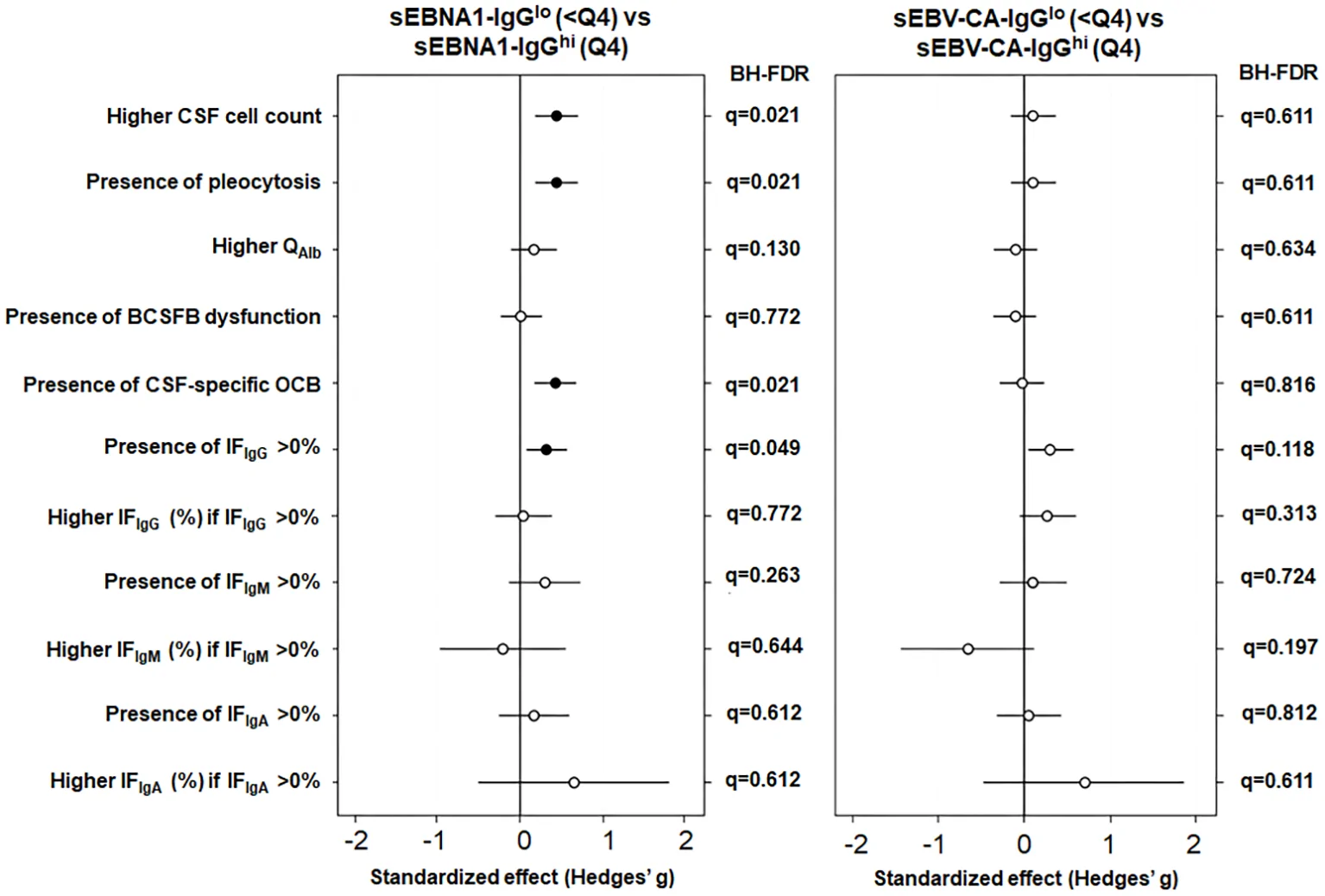
Association maps of basic parameters of CSF inflammation in MS patients with versus without high serum EBV-specific IgG levels (Q4 versus <Q4 levels). Forest-style summary of standardized differences between patients in the lower quartiles (<Q4) versus the highest quartile (Q4) of serum EBNA1-specific IgG levels (left panel, sEBNA1-IgG^lo^ vs sEBNA1-IgG^hi^) and serum EBV-CA-specific IgG levels (right panel, sEBV-CA-IgG^lo^ vs sEBV-CA-IgG^hi^). Points indicate Hedges’ g, i.e. the small-sample bias-corrected standardized mean difference between two groups for one outcome, after comparing Q4 levels as reference versus <Q4 levels; horizontal bars indicate 95% confidence intervals. The vertical line at g=0 indicates no difference between groups. Benjamini–Hochberg false discovery rate (BH-FDR)-adjusted q-values are specified in corresponding right-side column for each outcome. Filled points denote q<0.05 and open points denote q≥0.05. Positive g values indicate higher outcome values (or higher event probability for binary outcomes) in Q4 levels compared with <Q4 levels. Outcomes include (i) CSF parameters of cellular inflammation, i.e. higher CSF leukocyte count, and presence of pleocytosis (CSF leukocyte count >4 cells/µL); (ii) parameters of blood-CSF barrier dysfunction (BCSFB), i.e. higher Q_Alb_ (CSF/serum albumin quotient) and presence of BCSFB; and (iii) parameters of general intrathecal humoral immune activation, i.e. presence of CSF-specific OCB (oligoclonal bands type 2 or 3), presence of intrathecal fraction of IgG, IgM, or IgA >0% (presence of IF_IgG_, IF_IgM_, or IF_IgA_ >0%, i.e. intrathecal production of IgG, IgM, or IgA antibodies); and higher intensity of IF_IgG_ (if IF_IgG_ >0%), IF_IgM_ (if IF_IgM_ >0%), or IF_IgA_ (if IF_IgA_ >0%).

Dose-response analyses using continuous antibody concentrations showed broadly monotonic associations between increasing sEBNA1-IgG levels and basic markers of intrathecal immune activation, including CSF leukocyte count, presence of CSF-restricted OCB, and presence of intrathecal fraction of IgG (IF_IgG_) >0%, i.e. intrathecal IgG synthesis of total IgG according to Reiber (12) (**Figure 3**). In comparison, sEBV-CA-IgG concentrations displayed weaker and less consistent exposure-response patterns for CSF cell count outcomes, while associations with humoral intrathecal activity were more evident. Restricted cubic spline models yielded curves largely comparable to the primary log-linear models, with widening uncertainty at extreme antibody concentrations where observations were sparse (**Supplementary Figure 4**). To facilitate direct visual assessment of the empirical data underlying these model-based relationships, individual observed outcome values were additionally overlaid on the corresponding prediction curves (**Supplementary Figure 5**). Both, age at sampling and disease duration, did not influence the effect of serum EBV-specific IgG levels on above-mentioned basic cellular and humoral measures of CSF inflammation in this cohort, but the patients’ age and disease duration were probably too narrowly distributed to allow detection of any effects.

**Figure 3:**
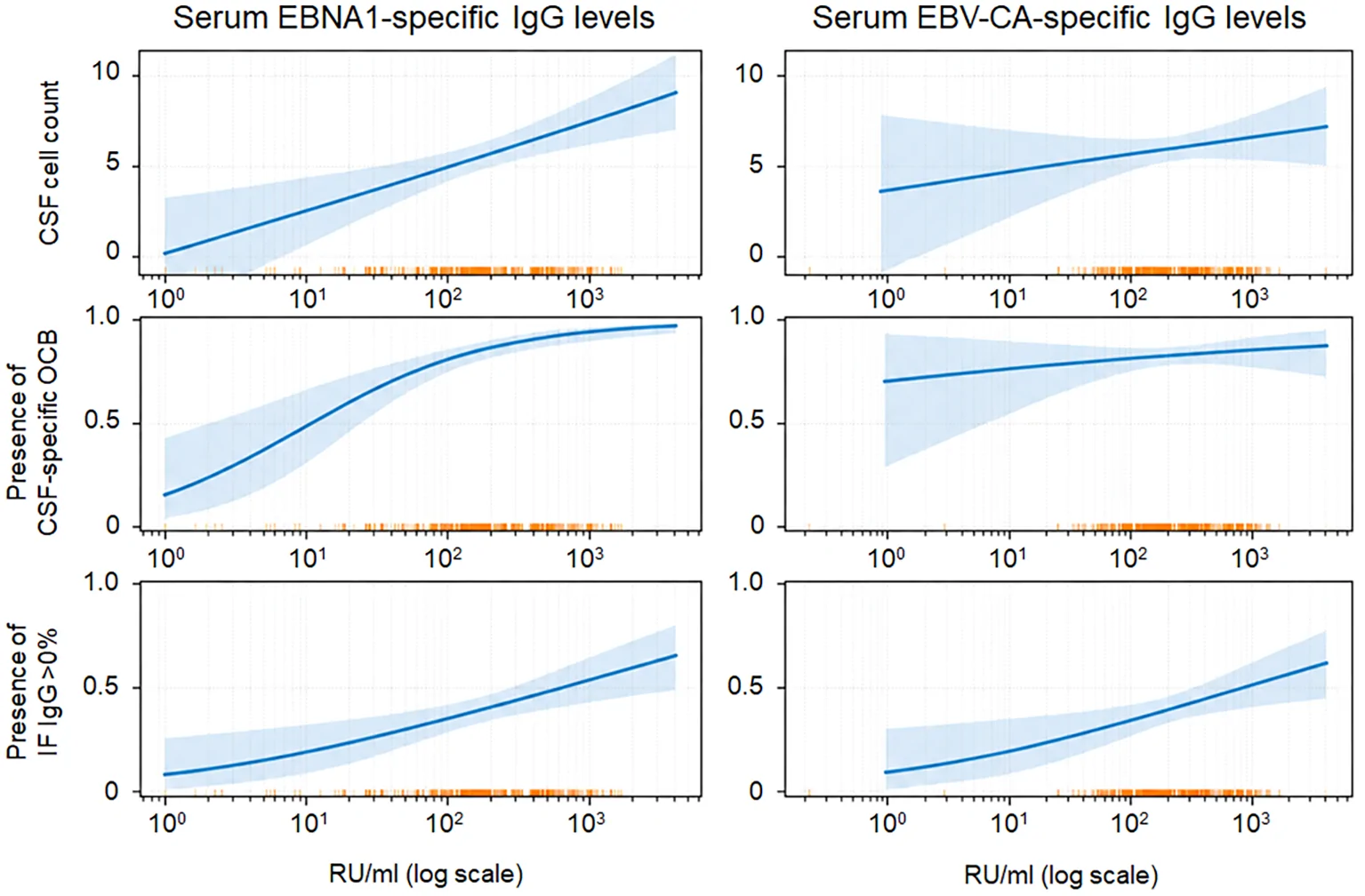
Dose–response relationships between absolute serum EBNA1- and EBV-CA-specific antibody concentrations and selected basic parameters of CSF inflammation. Panels show predicted associations for serum EBNA1-specific IgG (left column) and serum EBV-CA-specific IgG (right column), modeled as continuous exposures. The x-axis displays absolute antibody concentrations (RU/mL) on a log10 scale. Outcomes are shown as model-based predictions with 95% confidence intervals (shaded bands). The three outcomes were: (i) CSF leukocyte count (cells/µL; modeled on the log1p scale and back-transformed for plotting), (ii) presence of CSF-specific OCB (oligoclonal bands types 2/3; binary), and (iii) presence of IF_IgG_ >0% (intrathecal fraction of IgG >0%, i.e. intrathecal production of total IgG according to Reiber 1998; binary). For binary outcomes, curves represent the predicted probability. Short tick marks in orange along the x-axis (“rug”) indicate the distribution/density of observed serum EBNA1-specific and EBV-CA-specific antibody concentration values. All models used the available measurements for each outcome; exact number or measurements per panel corresponds to the underlying data availability described in **Table 1**.

When analyzing IF of total IgG, -IgA or -IgM, sEBV-IgG-related signals related stronger to the presence of intrathecal synthesis (IF>0%) than for intensity of intrathecal production of antibodies among patients with IF>0% (**Supplementary Table 6**, **7**). IF_IgG_ >0% was associated with higher sEBNA1-IgG levels (p=0.000504; FDR q=0.00202; Cliff’s δ=0.23) and higher sEBV-CA-IgG levels (p=0.0229; q=0.0458; δ=0.15) (**Supplementary Table 6**). For IF_IgM_ >0%, EBV-CA-CAI was higher in IF-positive patients (p=0.00291; q=0.0117; δ=0.36) (**Supplementary Table 6**). Among patients with IF >0%, only IF_IgG_ intensity correlated with EBV-CA-CAI (ρ=0.229; p=0.00414; q=0.0166); other intensity associations were not significant (**Supplementary Table 7**).

### 3.4 Intrathecal EBV-specific antibody production is uncommon in MS and not linked to serum EBV-specific IgG levels or basic CSF inflammation

Intrathecal EBV-specific antibody production was uncommon in this cohort, with EBNA1-CAI ≥1.5 in 18/309 patients (5.8%) and EBV-CA-CAI ≥1.5 in 22/309 patients (7.1%). Intrathecal EBV-specific IgG production was not associated with CSF leukocyte count, pleocytosis, Q_Alb_, blood-CSF barrier dysfunction, or presence of CSF-restricted OCB (all FDR q-values ≥0.844) (**Supplementary Table 8**).

In direct correlation analyses of absolute antibody concentrations, serum EBNA1-specific IgG levels were strongly correlated with the corresponding derived CSF concentrations (n=309; Spearman’s ρ=0.73, p<0.001), as were serum and CSF EBV-CA-specific IgG levels (n=308; ρ=0.76, p<0.001). In contrast, serum EBNA1-specific IgG levels did not significantly correlate with EBNA1-CAI (ρ=0.07, p=0.208), and serum EBV-CA-specific IgG levels did also not significantly correlate with EBV-CA-CAI (ρ=0.07, p=0.251).

To further examine the relationship between systemic and intrathecal EBV-specific humoral immunity, we compared the frequency of CAI positivity between patients with sEBV-IgG^hi^ levels and those with sEBV-IgG^lo^ levels (**Supplementary Table 9**). EBNA1-CAI positivity occurred in 4/77 (5.2%) patients with sEBNA1-IgG^hi^ levels and in 14/232 (6.0%) patients with sEBNA1-IgG^lo^ levels (OR = 0.85, 95% CI 0.27–2.67; p=1.0000; FDR q=1.0000). EBV-CA-CAI positivity was similarly distributed between these groups (6/77 [7.8%] versus 16/232 [6.9%]; OR = 1.14, 95% CI 0.43–3.03; p=0.7999; q=1.0000) (**Supplementary Table 9**).

Among patients grouped by sEBV-CA-specific IgG levels, EBNA1-CAI positivity was observed in 2/77 (2.6%) patients with sEBV-CA-IgG^hi^ levels and 16/232 (6.9%) patients with sEBV-CA-IgG^lo^ levels (OR = 0.36, 95% CI 0.08–1.60; p=0.2598; q=0.8944), whereas EBV-CA-CAI positivity was present in 7/77 (9.1%) and 15/232 (6.5%), respectively (OR = 1.45, 95% CI 0.57–3.69; p=0.4472; q=0.8944) (**Supplementary Table 9**). Similar to the overall group, the sex-stratified analyses also showed no significant signals (**Supplementary Table 9**).

Altogether, high systemic EBV-specific IgG levels were not accompanied by a higher frequency of intrathecal EBV-specific antibody production.

## 4 Discussion

In this study, we found that higher systemic EBV-specific seroreactivity, but not intrathecal EBV-specific antibody production, and rather sEBNA1-IgG^hi^ than sEBV-CA-IgG^hi^ levels were associated with a more inflammatory CSF profile regarding both general cellular and humoral immune activation in MS.

Our results are in accordance with two previous studies that have also shown an association of sEBNA1-IgG levels with intrathecal humoral immune activation as represented by intrathecal production of IgG or CSF-specific OCB in MS patients (9, 10). However, until now, an association of sEBNA1-IgG levels with CSF leukocyte numbers or an association of sEBV-CA-IgG levels with intrathecal production of IgG or CSF-specific OCB has not been described.

An interesting feature of our findings is the contrast between sEBNA1-IgG and sEBV-CA-IgG levels regarding effects on basic parameters of CSF inflammation. While sEBNA1-IgG^hi^ levels were robustly associated with CSF cellular inflammatory activity (CSF cell count and pleocytosis) and a key marker of intrathecal humoral activation (CSF-restricted OCB), sEBV-CA-IgG^hi^ levels showed weaker and less consistent associations with inflammatory endpoints. The most prominent sEBV-CA-IgG-related signal was a higher IgG index, suggesting that sEBV-CA-IgG levels may indicate elevated intrathecal humoral activity than with cellular CSF inflammation. This antigen-specific divergence may be caused by the different expression patterns and roles of EBNA1 and EBV-CA antigens during the viral lifecycle (25). EBNA1 is expressed continuously during both lytic replication and all latency programs, it elicits prominent chronic CD4+ T cell responses and its glycine-alanine repeat inhibits proteasomal degradation and MHC-I presentation and CD8+ T cell activation, whereas the expression of EBV-CA is restricted to the lytic phase prompting strong humoral responses but limited T cell immunity (26, 27). So, EBNA1- and EBV-CA-specific immunoreactivities capture different aspects of EBV-specific immune memory and may reflect distinct immunological “set points” shaped by infection history, expression and viral reactivation patterns, and/or host genetics. In MS, EBNA1-directed IgG responses have repeatedly been highlighted as particularly prominent (1, 16, 28), and our data add CSF correlates to this perspective. It is still not clear whether EBV-specific cellular and/or humoral immune reactions fuel the disease process or whether more tissue damage and release of viral antigens and autoantigens increase EBV-specific immunity, e.g. by cross-reactivity to autoantigens (5, 7, 29–31).

The dose-response analyses support the notion that there is a continuous relationship between sEBNA1-IgG levels and key CSF outcomes, even without using an arbitrarily chosen cutoff of Q4 versus <Q4 serum IgG levels. For sEBV-CA-IgG levels, continuous models were less consistent for inflammatory cell-count outcomes, again reinforcing that sEBNA1-IgG reactivity is the more coherent marker of the inflammatory CSF phenotype in this dataset.

Notably, as reported previously (32), intrathecal EBV-specific antibody production was uncommon and did not associate with basic CSF inflammatory measures, blood-CSF barrier markers, or presence of CSF-specific OCB. The low rate of intrathecal production of IgG against EBNA1 (5.8%) or EBV-CA (7.1%) in the present study fits with previous studies using commercial ELISA assays and Reiber’s formulas (12) for calculation of CAI’s and showing 2.0-10.0% prevalence of intrathecal EBNA1- or EBV-CA-specific IgG production among 653 MS patients (33–40). Other, usually smaller scaled studies using non-commercial in-house assays, EBV lysates from various sources and/or not applying Reiber’s formulas found more varying prevalences of intrathecal EBV-specific IgG production between 15.0% and 100% (38, 41–48). The relative lack of intrathecal EBV-specific antibody production in our and the above cited bigger studies is an important negative result and can be explained by the following scenario as outlined by Ruprecht and co-workers (38, 40): Acute primary EBV infection triggers a strong polyclonal activation of B cells in lymphoid tissue and peripheral blood. In individuals who later develop MS, polyspecific antibody-producing B lineage cells may enter the CNS predominantly in this early phase. Since the generation and increase of EBV-specific B cells is delayed after primary EBV infection, the circulating pool during early EBV infection is dominated by non-EBV-specific B lineage cells, which may preferentially invade the CNS and persist, establishing intrathecal polyspecific antibody production. This could also account for the higher prevalence of intrathecal production of measles (M)-, rubella (R)- and varicella zoster virus (Z)-specific antibodies (MRZ reaction, approx. 40%) compared to intrathecal EBV-specific antibody production (2-10%) in MS (21, 40). This scenario is also compatible with the view, that an active infection of the CNS with EBV could be less relevant in MS than peripheral EBV-induced immune dysregulation, which may be sufficient to trigger the disease process (38, 40).

The intrathecal fraction analyses further refine the interpretation that systemic EBV-related immune activity – reflected by serum titers – is the dominant correlate of CSF inflammation. EBV-related signals were generally stronger for the presence of intrathecal immunoglobulin synthesis (IF>0%) than for the intensity of IF among MS patients with IF >0%. Patients with intrathecal IgG synthesis had higher sEBNA1-IgG and sEBV-CA-IgG concentrations, whereas correlations between IF intensity and sEBV-IgG levels were limited, with a modest association observed only between IF_IgG_ intensity and EBV-CA-CAI. This pattern suggests that systemic EBV-specific seroreactivity is linked to whether intrathecal synthesis is present at all, consistent with a broader immunological activation phenotype, while the quantitative “strength” of intrathecal antibody synthesis within the positive subgroup may be influenced by additional factors (timing of sampling, disease stage, treatment exposure, assay variability, and biological heterogeneity). The weaker intensity findings for intrathecal IgM and IgA production likely also reflect smaller sample sizes and reduced statistical power.

Several mechanisms could explain why high sEBNA1-IgG titers co-occur with inflammatory CSF findings in MS. One possibility is that high sEBV-IgG titers reflect a EBV-induced systemic expansion and activation of memory B-cells, which migrate to the CNS and lead to CNS-directed inflammation and intrathecal cellular and humoral immune activation (49, 50). Alternatively, high sEBV-IgG titers and general inflammatory CSF alterations in MS may be confounded by host factors that shape both antiviral immunity and MS immunopathology, such as HLA background, B cell repertoire features, and/or immune regulation set points. Indeed, genetic and sex determinants, predominantly HLA-DR15 and female sex, have been associated with increased sEBNA1-IgG titers, as well as with intrathecal production of total IgG, CSF-specific OCB and MRZ reaction (51–59). However, recent data support the first scenario, in which EBV in peripheral lymphoid tissues, such as deep cervical lymph nodes, primes and drives a pro-inflammatory B-cell program, especially in memory-like and T-bet+ CD11c+ CXCR3+ B cell subsets, which promote secretion of pro-inflammatory cytokines, show broad antigen-presenting and T cell-stimulating capabilities and facilitate formation of spontaneous germinal centers (60–63). Such EBV-shaped and expanded oligoclonal T-bet+ CXCR3+ B cells can home to the CNS, colonize meninges and submeningeal spaces, and attract and amplify pro-inflammatory T cells to create locoregional self-sustaining B-T cell clusters (49, 61). In genetically susceptible people, especially in HLA-DR15+ individuals, EBV may reprogram transcriptomes and antigen processing in B cells, especially HLA-DR15+ B cells, so that they preferentially present specific CNS-derived myelin basic protein peptides, and these very peptides are found on EBV-infected B cells and MS brain tissue, but not on primary B cells or thymic tissue (64). Hence, EBV-infected B cells in the periphery can expand T cells reactive against these myelin basic protein peptides, and these T cells encounter their cognate antigen at high density only in the CNS, where myelin-derived proteins are enriched and accessible, creating a CNS-biased loop in MS patients (64). In addition, EBNA1-specific T cells may cross-react to CNS-associated autoantigens such as MBP, anoctamin-2 and alpha-crystallin B (5, 30, 31, 65). Our study was not designed to distinguish among these mechanisms, but the combined pattern (stronger serum signals than intrathecal EBV-specific CAI signals, and stronger associations for EBNA1- than EBV-CA-specific antibodies) is consistent with an outside-in model, in which an environmental trigger such as EBV outside of the CNS induces CNS-directed autoimmune processes leading to MS, as opposed to an inside-out model, in which the pathogenic autoimmune processes are primarily initiated by CNS-internal events.

Strengths of this work include the relatively large cohort with paired serum/CSF sampling, the use of complementary exposure definitions (high serum levels, intrathecal CAI positivity), and the systematic evaluation of multiple CSF parameters (cellular inflammation, barrier function, intrathecal humoral activation). Importantly, we handled multiplicity using FDR within predefined endpoint families and complemented categorical comparisons with dose-response modeling and minimal adjusted regression analyses, improving robustness and interpretability.

The study also has limitations. First, its cross-sectional design does not allow for conclusions about causal relationships and does not account for temporal relationships: high serum EBNA1-specific antibody titers may precede, accompany, or follow intrathecal inflammation. However, very high levels of these antibodies develop already early after primary EBV infection and many years before MS onset (6). Second, although we used age- and sex-adjusted models for core inflammatory outcomes, residual confounding (e.g., treatment status, recent relapse activity, or time from onset to sampling) may influence associations. Third, some outcomes (notably IgM/IgA-related measures and IF intensity analyses) were available only in subsets, limiting power and precision. Fourth, intrathecal EBV-specific CAI positivity was rare, limiting sensitivity to detect small differences; however, the absence of even directional trends across several CSF endpoints argues against large effects. Fifth, our study did not include healthy control CSF samples, and our analysis was limited to EBNA1- and EBVCA-specific CAIs, precluding comparison with physiological levels and assessment of responses to other EBV proteins. Furthermore, although assay sensitivity is expected to be comparable for serum and CSF samples, thereby minimizing its impact on the CAI-based assessment, very low-abundance antibodies produced specifically within the CNS may remain below the detection threshold and should therefore be considered when interpreting our findings.

In summary, our data show that high serum EBV-specific IgG levels, particularly serum EBNA1-reactive IgG levels, are associated with a more inflammatory CSF phenotype and stronger intrathecal humoral activation in MS, whereas intrathecal EBV-specific antibody production is uncommon and not associated with general markers of CSF inflammation. These findings support the concept that systemic EBV-specific immune reactivities are linked to general intrathecal immune activity in MS, and they motivate future longitudinal and mechanistic studies to clarify whether EBNA1-directed seroreactivity is a marker of disease-relevant immune activation, a proxy for upstream host determinants, or part of a causal pathway influencing CNS inflammation.

## Data Availability

The raw data supporting the conclusions of this article will be made available by the authors, without undue reservation.
