# Supplementary material for: High EBV-reactive IgG levels in serum are linked to typical inflammatory cerebrospinal fluid features in multiple sclerosis
Source: Front Immunol. 2026 Oct 5;17:1929166. doi: 10.3389/fimmu.2026.1929166 (PMC13677834; doi:10.3389/fimmu.2026.1929166)
Supplement: Supplementary file 1 [file DataSheet1.pdf]

Supplementary Material

High EBV-reactive IgG levels in serum are linked to typical inflammatory cerebrospinal fluid features in multiple sclerosis

Stephan Neidhart [1,2,3], Klara Asplund Högelin [4,5], Marc Hilty [1,2,3], Benjamin Vlad [1,6], Mario Ziegler [1], Mohsen Khademi [4], Andreas Lutterotti [1,2,3,7], Roland Martin [1,7,8,9], Faiez Al Nimer [4] *, Ilijas Jelcic [1,2,3] *

[1] Neuroimmunology and Multiple Sclerosis Research Section, Department of Neurology, University Hospital Zurich and University of Zurich, Zurich, Switzerland

[2] Department of Neurology, Hirslanden Klinik Zurich, Zurich, Switzerland

[3] Center for Multiple Sclerosis and Clinical Neuroimmunology, Bellevue Medical Group, Zurich, Switzerland

[4] Center for Molecular Medicine, Neuroimmunology Unit, Department of Clinical Neuroscience, Karolinska Institutet, Stockholm, Sweden

[5] Department of Laboratory Medicine, Karolinska Institutet, Stockholm, Sweden.

[6] Section Translational Neuroimmunology, Department of Neurology, Jena University Hospital, Jena, Germany

[7] Cellerys AG, Schlieren, Switzerland

[8] Institute of Experimental Immunology, University of Zurich, Zurich, Switzerland

[9] Center for Molecular Medicine, Therapeutic Immune Design Unit, Department of Clinical Neuroscience, Department of Clinical Neuroscience, Karolinska Institutet, Stockholm, Sweden

*** Correspondence:**

Ilijas Jelcic, MD, University of Zurich, Zurich, Switzerland, e-mail: ilijas.jelcic@uzh.ch

Faiez Al Nimer, MD, PhD, Center for Molecular Medicine, Neuroimmunology Unit, Department of Clinical Neuroscience, Karolinska Institutet, Stockholm, Sweden, faiez.al.nimer@ki.se

*both authors contributed equally

Supplementary Table 1. Differences in basic CSF parameters among female MS patients with and without high serum EBNA1-specific IgG levels (Q4 versus <Q4). High serum EBNA1-specific IgG levels (Q4, sEBNA-IgG^{hi}) were defined as >336 RU/mL, and serum EBNA1-specific IgG levels <Q4 (sEBNA1-IgG^{lo}) were <336 RU/mL. Significant findings are marked in bold.

**Outcome in female MS patients**	**sEBNA1-IgG^{hi} (Q4)**	**sEBNA1-IgG^{lo} (<Q4)**	**p-value**	**FDR q-value**
n/N (%)	55/213 (25.8%)	158/213 (74.2%)	–	–
CSF leukocyte count, cells/µL, median (IQR)	8.0 (2.0–12.5)	4.0 (2.0–8.0)	0.0043	**0.0278**
Patients with pleocytosis, n/N (%)	32/55 (58.2%)	63/158 (39.9%)	0.0186	**0.0606**
Q_{Alb}, median (IQR)	4.7 (3.7–5.4)	4.2 (3.3–5.5)	0.2031	0.3772
BCSFB dysfunction, n/N (%)	8/55 (14.5%)	32/158 (20.3%)	0.3506	0.5697
CSF-specific OCB, n/N (%)	53/55 (96.4%)	122/158 (77.2%)	0.0014	**0.0182**
IgG index, median (IQR)	0.8 (0.6–1.1)	0.7 (0.5–1.0)	0.0743	0.1933
Elevated IgG index (>0.7), n/N (%)	33/55 (60.0%)	76/158 (48.1%)	0.1284	0.2782
Intrathecal synthesis of total IgG (Reiber's IF_{IgG} >0%), n/N (%)	39/55 (70.9%)	80/158 (50.6%)	0.0091	**0.0394**
Intensity of IF_{IgG} (%), median (IQR), among patients with IF_{IgG}>0% (n)*	35.6 (16.5–55.5) (n=40)	34.9 (16.2–47.2) (n=83)	0.8607	0.8784
Intrathecal synthesis of total IgM (Reiber's IF_{IgM} >0%), n/N (%)	4/17 (23.5%)	14/71 (19.7%)	0.7428	0.8779
Intensity of IF_{IgM} (%), median (IQR), among patients with IF_{IgM}>0% (n)*	49.7 (41.3–54.1) (n=4)	51.4 (25.9–63.2) (n=14)	0.8784	0.8784
Intrathecal synthesis of total IgA (Reiber's IF_{IgA}), n/N (%)	2/17 (11.8%)	5/71 (7.0%)	0.6165	0.8014
Intensity of IF_{IgA} (%), median (IQR), among patients with IF_{IgA}>0% (n)*	50.8 (34.8–66.7) (n=2)	23.3 (2.5–25.3) (n=5)	0.5714	0.8014

Continuous variables are reported as median (IQR) (with outcome-specific n); categorical variables as n/N (%). P-values are from Mann–Whitney U tests or chi-square/Fisher's exact tests as appropriate. Within each table, p-values across reported outcomes were adjusted using Benjamini–Hochberg; false discovery rate (FDR)-adjusted q-values are shown. Intrathecal fraction (IF) summaries are restricted to patients with IF >0%.
Abbreviations: BCSFB = blood–CSF barrier; CSF = cerebrospinal fluid; IF = intrathecal fraction; OCB = oligoclonal bands; Q_{Alb} = CSF/serum albumin quotient.
*n = number of patients with $IF_{IgG/IgM/IgA}$>0%

Supplementary Table 2. Differences in basic CSF parameters among male MS patients with and without high serum EBNA1-specific IgG levels (Q4 versus <Q4). High serum EBNA1-specific IgG levels (Q4, sEBNA-IgG^{hi}) were defined as >336 RU/mL, and serum EBNA1-specific IgG levels <Q4 (sEBNA1-IgG^{lo}) were <336 RU/mL. Significant findings are marked in bold.

**Outcome in male MS patients**	**sEBNA1-IgG^{hi} (Q4)**	**sEBNA1-IgG^{lo} (<Q4)**	**p-value**	**FDR q-value**
n/N (%)	24/103 (23.3%)	79/103 (76.7%)	–	–
CSF leukocyte count, cells/µL, median (IQR)	4.5 (1.8–8.2)	2.0 (2.0–6.0)	0.4147	0.7143
Patients with pleocytosis, n/N (%)	12/24 (50.0%)	26/79 (32.9%)	0.1286	0.4181
Q_{Alb}, median (IQR)	6.8 (4.2–7.9)	5.2 (4.2–7.1)	0.1133	0.4181
BCSFB dysfunction, n/N (%)	14/24 (58.3%)	30/79 (38.0%)	0.0774	0.4181
CSF-specific OCB, n/N (%)	21/24 (87.5%)	66/79 (83.5%)	0.7577	0.9846
IgG index, median (IQR)	0.7 (0.6–0.8)	0.6 (0.5–0.7)	0.2629	0.6837
Elevated IgG index (>0.7), n/N (%)	9/24 (37.5%)	23/79 (29.1%)	0.4369	0.7143
Intrathecal synthesis of total IgG (Reiber's IF_{IgG} >0%), n/N (%)	8/24 (33.3%)	26/79 (32.9%)	0.9693	0.9846
Intensity of IF_{IgG} (%), median (IQR), among patients with IF_{IgG}>0% (n)*	26.5 (16.7–49.7) (n=8)	28.8 (13.7–51.7) (n=27)	0.9846	0.9846
Intrathecal synthesis of total IgM (Reiber's IF_{IgM} >0%), n/N (%)	5/10 (50.0%)	10/43 (23.3%)	0.1235	0.4181
Intensity of IF_{IgM} (%), median (IQR), among patients with IF_{IgM}>0% (n)*	29.1 (17.4–78.2) (n=5)	59.9 (38.4–74.1) (n=10)	0.4396	0.7143
Intrathecal synthesis of total IgA (Reiber's IF_{IgA}), n/N (%)	2/10 (20.0%)	6/43 (14.0%)	0.6364	0.9193
Intensity of IF_{IgA} (%), median (IQR), among patients with IF_{IgA}>0% (n)*	44.0 (28.7–59.2) (n=2)	32.7 (21.4–36.2) (n=6)	0.8571	0.9846

Continuous variables are reported as median (IQR) (with outcome-specific n); categorical variables as n/N (%). P-values are from Mann–Whitney U tests or chi-square/Fisher's exact tests as appropriate. Within each table, p-values across reported outcomes were adjusted using Benjamini–Hochberg; false discovery rate (FDR)-adjusted q-values are shown. Intrathecal fraction (IF) summaries are restricted to patients with IF >0%.
Abbreviations: BCSFB = blood–CSF barrier; CSF = cerebrospinal fluid; IF = intrathecal fraction; OCB = oligoclonal bands; Q_{Alb} = CSF/serum albumin quotient.
*n = number of patients with $IF_{IgG/IgM/IgA}$>0%

Supplementary Table 3. Differences in basic CSF parameters among female MS patients with and without very high serum EBV-CA-specific IgG levels (Q4 versus <Q4). High serum EBV-CA-specific IgG levels (Q4, sEBV-CA-IgG^{hi}) were defined as >372 RU/mL, and serum EBV-CA-specific IgG levels <Q4 (sEBV-CA-IgG^{lo}) were <372 RU/mL. Significant findings are marked in bold.

**Outcome in female MS patients**	**sEBV-CA-IgG^{hi} (Q4)**	**sEBV-CA IgG^{lo} (<Q4)**	**p-value**	**FDR q-value**
n/N (%)	64/213 (30.0%)	149/213 (70.0%)	–	–
CSF leukocyte count, cells/µL, median (IQR)	4.0 (2.0–8.0)	4.0 (2.0–10.0)	0.8176	1.0000
Patients with pleocytosis, n/N (%)	29/64 (45.3%)	66/149 (44.3%)	0.8911	1.0000
Q_{Alb}, median (IQR)	4.3 (3.3–5.5)	4.3 (3.4–5.5)	0.8357	1.0000
BCSFB dysfunction, n/N (%)	12/64 (18.8%)	28/149 (18.8%)	0.9943	1.0000
CSF-specific OCB, n/N (%)	52/64 (81.2%)	123/149 (82.6%)	0.8202	1.0000
IgG index, median (IQR)	0.8 (0.6–1.1)	0.7 (0.5–1.0)	0.0394	0.2561
Elevated IgG index (>0.7), n/N (%)	41/64 (64.1%)	68/149 (45.6%)	0.0137	0.1775
Intrathecal synthesis of total IgG (Reiber's IF_{IgG} >0%), n/N (%)	40/64 (62.5%)	79/149 (53.0%)	0.2014	0.6547
Intensity of IF_{IgG} (%), median (IQR), among patients with IF_{IgG}>0% (n)*	39.4 (18.7–50.4) (n=42)	31.4 (16.1–47.0) (n=81)	0.3832	0.8303
Intrathecal synthesis of total IgM (Reiber's IF_{IgM} >0%), n/N (%)	5/25 (20.0%)	13/63 (20.6%)	0.9469	1.0000
Intensity of IF_{IgM} (%), median (IQR), among patients with IF_{IgM}>0% (n)*	28.0 (25.2–46.7) (n=5)	54.3 (28.2–65.7) (n=13)	0.1172	0.5078
Intrathecal synthesis of total IgA (Reiber's IF_{IgA}), n/N (%)	2/25 (8.0%)	5/63 (7.9%)	1.0000	1.0000
Intensity of IF_{IgA} (%), median (IQR), among patients with IF_{IgA}>0% (n)*	52.9 (38.1–67.8) (n=2)	18.9 (2.5–25.3) (n=5)	0.3810	0.8303

Continuous variables are reported as median (IQR) (with outcome-specific n); categorical variables as n/N (%). P-values are from Mann–Whitney U tests or chi-square/Fisher's exact tests as appropriate. Within each table, p-values across reported outcomes were adjusted using Benjamini–Hochberg; false discovery rate (FDR)-adjusted q-values are shown. Intrathecal fraction (IF) summaries are restricted to patients with IF >0%.
Abbreviations: BCSFB = blood–CSF barrier; CSF = cerebrospinal fluid; IF = intrathecal fraction; OCB = oligoclonal bands; Q_{Alb} = CSF/serum albumin quotient.
*n = number of patients with $IF_{IgG/IgM/IgA}$>0%

Supplementary Table 4. Differences in basic CSF parameters among male MS patients with and without very high serum EBV-CA-specific IgG levels (Q4 versus <Q4). High serum EBV-CA-specific IgG levels (Q4, sEBV-CA-IgG^{hi}) were defined as >372 RU/mL, and serum EBV-CA-specific IgG levels <Q4 (sEBV-CA-IgG^{lo}) were <372 RU/mL. Significant findings are marked in bold.

**Outcome in male MS patients**	**sEBV-CA-IgG^{hi} (Q4)**	**sEBV-CA IgG^{lo} (<Q4)**	**p-value**	**FDR q-value**
n/N (%)	14/103 (7.7%)	89/103 (86.4%)	–	–
CSF leukocyte count, cells/µL, median (IQR)	5.0 (1.2–7.0)	3.0 (2.0–6.0)	0.7264	0.9443
Patients with pleocytosis, n/N (%)	7/14 (50.0%)	31/89 (34.8%)	0.2742	0.6063
Q_{Alb}, median (IQR)	5.6 (4.5–6.8)	5.5 (4.0–7.3)	0.6476	0.9354
BCSFB dysfunction, n/N (%)	6/14 (42.9%)	38/89 (42.7%)	0.9910	1.0000
CSF-specific OCB, n/N (%)	12/14 (85.7%)	75/89 (84.3%)	1.0000	1.0000
IgG index, median (IQR)	0.7 (0.6–1.0)	0.6 (0.5–0.7)	0.1826	0.6063
Elevated IgG index (>0.7), n/N (%)	7/14 (50.0%)	25/89 (28.1%)	0.1238	0.6063
Intrathecal synthesis of total IgG (Reiber's IF_{IgG} >0%), n/N (%)	6/14 (42.9%)	28/89 (31.5%)	0.5417	0.9354
Intensity of IF_{IgG} (%), median (IQR), among patients with IF_{IgG}>0% (n)*	43.7 (32.5–52.7) (n=6)	22.6 (9.5–49.3) (n=29)	0.1587	0.6063
Intrathecal synthesis of total IgM (Reiber's IF_{IgM} >0%), n/N (%)	4/9 (44.4%)	11/44 (25.0%)	0.2520	0.6063
Intensity of IF_{IgM} (%), median (IQR), among patients with IF_{IgM}>0% (n)*	39.6 (13.4–65.7) (n=4)	58.1 (31.5–78.7) (n=11)	0.2799	0.6063
Intrathecal synthesis of total IgA (Reiber's IF_{IgA}), n/N (%)	2/9 (22.2%)	6/44 (13.6%)	0.6109	0.9354
Intensity of IF_{IgA} (%), median (IQR), among patients with IF_{IgA}>0% (n)*	44.0 (28.7–59.2) (n=2)	32.7 (21.4–36.2) (n=6)	0.8571	1.0000

Continuous variables are reported as median (IQR) (with outcome-specific n); categorical variables as n/N (%). P-values are from Mann–Whitney U tests or chi-square/Fisher's exact tests as appropriate. Within each table, p-values across reported outcomes were adjusted using Benjamini–Hochberg; false discovery rate (FDR)-adjusted q-values are shown. Intrathecal fraction (IF) summaries are restricted to patients with IF >0%.
Abbreviations: BCSFB = blood–CSF barrier; CSF = cerebrospinal fluid; IF = intrathecal fraction; OCB = oligoclonal bands; Q_{Alb} = CSF/serum albumin quotient.
*n = number of patients with $IF_{IgG/IgM/IgA}$>0%

Supplementary Table 5. Minimal age- and sex-adjusted models linking serum EBV-specific IgG reactivity to basic parameters of cellular CSF inflammation. Linear models use log1p CSF cell count; logistic models use pleocytosis (>4 cells/µL). Predictors are per 10-fold increase in serum EBNA-specific IgG levels (sEBNA1-IgG) or serum EBVA-CA-specific IgG levels (sEBV-CA-IgG) log10 RU/mL. Robust regression (Huber) yielded similar estimates for the linear model.

**Outcome**	**Model**	**Predictor (per 10-fold increase)**	**Estimate**	**95% CI**	**p-value**
CSF cell count (log1p)	Linear (OLS), adjusted for age+sex	sEBNA1-IgG (log10 RU/mL)	β=0.405	0.217–0.592	**2.86e-5**
CSF cell count (log1p)	Linear (OLS), adjusted for age+sex	sEBV-CA-IgG (log10 RU/mL)	β=0.227	-0.019–0.473	0.070
Pleocytosis (>4 cells/µL)	Logistic, adjusted for age+sex	sEBNA1-IgG (log10 RU/mL)	OR=2.73	1.59–4.71	**2.83e-4**
Pleocytosis (>4 cells/µL)	Logistic, adjusted for age+sex	sEBV-CA-IgG (log10 RU/mL)	OR=1.92	1.00–3.67	0.050

Abbreviations: CI = confidence interval; CSF = cerebrospinal fluid; EBV-CA = EBV capsid antigen; OLS = ordinary least squares; OR = odds ratio; RU/mL = relative units per millilitre.

Supplementary Table 6. Associations of intrathecal immunoglobulin synthesis according to Reiber (1998) with serum EBV-specific IgG levels. Serum EBNA1-specific IgG (sEBNA-IgG) levels and serum EBV-CA-specific IgG (sEBV-CA-IgG) levels are compared between patients with intrathecal production of antibodies (intrathecal fraction, IF >0%) and without intrathecal production of antibodies (IF ≤0%) using Mann-Whitney U test. EBV parameters are median (IQR). Cliff's δ is shown as an effect size between -1 and +1 with 0.0 indicating complete overlap (no effect), 0.11 ≤ |δ| < 0.28 indicating small effect, 0.28 ≤ |δ| < 0.43 indicating medium effect, |δ| ≥ 0.43 indicating large effects. A positive δ value indicates the first group generally has higher values; a negative δ value indicates the second group has higher values.

IF class	EBV parameter	Patients with IF >0%		Patients with IF ≤0%		p-value	FDR q-value	Cliff's δ
		n	EBV parameter	n	EBV parameter			
IF_{IgG}	sEBNA1-IgG, RU/ml	158	187.66 (137.83–453.59)	158	156.66 (83.77–277.44)	0.000504	**0.00202**	0.23
IF_{IgG}	sEBV-CA-IgG, RU/ml	158	198.09 (139.05–434.67)	157	171.14 (131.48–294.94)	0.0229	**0.0458**	0.15
IF_{IgG}	EBNA1-CAI	155	0.48 (0.38–0.65)	154	0.53 (0.39–0.68)	0.332	0.36	-0.06
IF_{IgG}	EBV-CA-CAI	155	0.59 (0.44–0.74)	154	0.61 (0.48–0.77)	0.36	0.36	-0.06
IF_{IgM}	sEBNA1-IgG, RU/ml	33	171.44 (144.70–434.18)	108	165.18 (111.44–189.36)	0.185	0.247	0.15
IF_{IgM}	sEBV-CA-IgG, RU/ml	33	169.57 (143.11–387.34)	108	167.41 (137.97–199.19)	0.71	0.71	0.04
IF_{IgM}	EBNA1-CAI	30	0.57 (0.52–0.76)	104	0.54 (0.43–0.73)	0.123	0.246	0.19
IF_{IgM}	EBV-CA-CAI	30	0.74 (0.58–1.04)	104	0.60 (0.49–0.70)	0.00291	**0.0117**	0.36
IF_{IgA}	sEBNA1-IgG, RU/ml	15	155.85 (107.31–349.26)	126	170.20 (125.58–190.72)	0.706	0.706	0.06
IF_{IgA}	sEBV-CA-IgG, RU/ml	15	169.60 (146.12–351.62)	126	167.41 (137.79–199.28)	0.481	0.641	0.11
IF_{IgA}	EBNA1-CAI	14	0.58 (0.54–0.82)	120	0.55 (0.44–0.72)	0.265	0.531	0.18
IF_{IgA}	EBV-CA-CAI	14	0.67 (0.62–0.77)	120	0.61 (0.50–0.77)	0.147	0.531	0.24

Abbreviations: IF = intrathecal fraction; RU/mL = relative units per millilitre; CAI = CSF/serum antibody index; FDR = false discovery rate.

Supplementary Table 7. Correlations of intensity of intrathecal immunoglobulin synthesis according to Reiber (1998) with serum EBV-specific IgG levels. Spearman correlations between intensity (%) of IF of total IgG (IF_{IgG}), of total IgM (IF_{IgM}) or total IgA (IF_{IgA}) and serum EBV-specific IgG levels among patients with IF >0%. Within each IF class (IF_{IgG}, IF_{IgM}, IF_{IgA}), p-values across EBV parameters were adjusted using Benjamini-Hochberg false discovery rate (FDR) and q-values are reported.

**IF class**	**EBV parameter**	**Patients with IF >0% (n)**	**Spearman ρ**	**p-value**	**FDR q-value**
IF_{IgG}	sEBNA1-IgG, RU/ml	158	0.034	0.672	0.672
IF_{IgG}	sEBV-CA-IgG, RU/ml	158	0.116	0.147	0.295
IF_{IgG}	EBNA1-CAI	155	0.046	0.568	0.672
IF_{IgG}	EBV-CA-CAI	155	0.229	0.00414	**0.0166**
IF_{IgM}	sEBNA1-IgG, RU/ml	33	0.032	0.859	0.859
IF_{IgM}	sEBV-CA-IgG, RU/ml	33	-0.260	0.144	0.578
IF_{IgM}	EBNA1-CAI	30	-0.120	0.527	0.801
IF_{IgM}	EBV-CA-CAI	30	-0.099	0.601	0.801
IF_{IgA}	sEBNA1-IgG, RU/ml	15	0.286	0.302	0.591
IF_{IgA}	sEBV-CA-IgG, RU/ml	15	0.400	0.14	0.558
IF_{IgA}	EBNA1-CAI	14	-0.223	0.444	0.591
IF_{IgA}	EBV-CA-CAI	14	-0.112	0.703	0.703

Abbreviations: IF = intrathecal fraction; EBV-CA = EBV capsid antigen; RU/mL = relative units per millilitre; CAI = CSF/serum antibody index; FDR = false discovery rate.

Supplementary Table 8. Differences in basic CSF parameters among MS patients with and without intrathecal EBV-specific IgG production. A) MS patients with and without intrathecal EBNA1-specific IgG production (EBNA1-CAI ≥1.5). B) MS patients with and without intrathecal EBV-CA-specific IgG production (EBV-CA-CAI ≥1.5).

**A) Intrathecal EBNA1-specific IgG production (EBNA1-CAI ≥1.5)**				
**Outcome**	**EBNA1-CAI ≥1.5**	**EBNA1-CAI <1.5**	**p-value**	**FDR q-value**
n/N (%)	18/309 (5.8%)	291/309 (94.2%)	–	–
CSF leukocyte count, cells/µL, median (IQR)	3.0 (1.0–8.2)	4.0 (2.0–8.0)	0.6114	0.8442
Patients with pleocytosis, n/N (%)	7/18 (38.9%)	120/291 (41.2%)	0.8442	0.8442
Q_{Alb}, median (IQR)	4.49 (3.67–4.76)	4.59 (3.60–6.26)	0.4605	0.8442
Patients with BCSFB dysfunction, n/N (%)	4/18 (22.2%)	77/291 (26.5%)	0.7899	0.8442
Patients with CSF-restricted OCB, n/N (%)	16/18 (88.9%)	239/291 (82.1%)	0.7487	0.8442
**B) Intrathecal EBV-CA-specific IgG production (EBV-CA CAI ≥1.5)**				
**Outcome**	**EBV-CA-CAI ≥1.5**	**EBV-CA-CAI <1.5**	**p-value**	**FDR q-value**
n/N (%)	22/309 (7.1%)	287/309 (92.9%)	–	–
CSF leukocyte count, cells/µL, median (IQR)	3.5 (1.2–9.2)	4.0 (2.0–8.0)	0.8098	1
Patients with pleocytosis, n/N (%)	10/22 (45.5%)	117/287 (40.8%)	0.6667	1
Q_{Alb}, median (IQR)	4.61 (4.14–5.74)	4.50 (3.60–6.20)	0.5294	1
Patients with BCSFB dysfunction, n/N (%)	6/22 (27.3%)	75/287 (26.1%)	0.9067	1
Patients with CSF-restricted OCB, n/N (%)	18/22 (81.8%)	237/287 (82.6%)	1	1

Continuous variables are median (IQR); categorical variables are n/N %. P-values are from Mann–Whitney U tests or chi-square/Fisher tests as appropriate. Within each panel (A or B), p-values across outcomes were adjusted using Benjamini–Hochberg; FDR q-values are reported.
Abbreviations: CAI = CSF/serum antibody index; OCB = oligoclonal bands; Q_{Alb} = CSF/serum albumin quotient.

Supplementary Table 9. Differences in prevalences of intrathecal EBNA1-specific IgG production (EBNA1-CAI ≥1.5) and intrathecal EBV-CA-specific IgG production (EBV-CA-CAI ≥1.5) among A) overall MS patients, B) female MS patients and C) male MS patients, each with and without high serum EBV-specific IgG levels, i.e. sEBV-IgG^{hi} (Q4) versus sEBV-IgG^{lo} (<Q4). sEBNA-IgG^{hi} were defined as >336 RU/mL, and sEBV-CA-IgG^{hi} were defined as >372 RU/mL.

Outcome	sEBV-IgG^{hi} (Q4), n/N (%)	sEBV-IgG^{lo} (<Q4), n/N (%)	OR (95% CI)	p-value	FDR q-value
**A) Overall cohort**					
- Effects of sEBNA1-IgG^{hi} (Q4) versus sEBNA1-IgG^{lo} (<Q4)					
EBNA1-CAI ≥1.5	4/77 (5.2%)	14/232 (6.0%)	0.85 (0.27–2.67)	1.0000	1.0000
EBV-CA-CAI ≥1.5	6/77 (7.8%)	16/232 (6.9%)	1.14 (0.43–3.03)	0.7999	1.0000
- Effects of sEBV-CA-IgG^{hi} (Q4) versus sEBV-CA-IgG^{lo} (<Q4)					
EBNA1-CAI ≥1.5	2/77 (2.6%)	16/232 (6.9%)	0.36 (0.08–1.60)	0.2598	0.8944
EBV-CA-CAI ≥1.5	7/77 (9.1%)	15/232 (6.5%)	1.45 (0.57–3.69)	0.4472	0.8944
**B) Females**					
- Effects of sEBNA1-IgG^{hi} (Q4) versus sEBNA1-IgG^{lo} (<Q4)					
EBNA1-CAI ≥1.5	2/55 (3.6%)	11/154 (7.1%)	0.49 (0.11–2.29)	0.5208	0.6944
EBV-CA-CAI ≥1.5	4/55 (7.3%)	11/154 (7.1%)	1.02 (0.31–3.35)	1.0000	1.0000
- Effects of sEBV-CA-IgG^{hi} (Q4) versus sEBV-CA-IgG^{lo} (<Q4)					
EBNA1-CAI ≥1.5	1/64 (1.6%)	12/145 (8.3%)	0.18 (0.02–1.38)	0.1154	0.4616
EBV-CA-CAI ≥1.5	7/64 (10.9%)	8/145 (5.5%)	2.10 (0.73–6.07)	0.2424	0.4848
**C) Males**					
- Effects of sEBNA1-IgG^{hi} (Q4) versus sEBNA1-IgG^{lo} (<Q4)					
EBNA1-CAI ≥1.5	2/22 (9.1%)	3/78 (3.8%)	2.50 (0.39–15.99)	0.3028	0.6470
EBV-CA-CAI ≥1.5	2/22 (9.1%)	5/78 (6.4%)	1.46 (0.26–8.10)	0.6470	0.6470
- Effects of sEBV-CA-IgG^{hi} (Q4) versus sEBV-CA-IgG^{lo} (<Q4)					
EBNA1-CAI ≥1.5	1/13 (7.7%)	4/87 (4.6%)	1.73 (0.18–16.79)	0.5092	0.6470
EBV-CA-CAI ≥1.5	0/13 (0.0%)	7/87 (8.0%)	0.40 (0.02–7.37)	0.5899	0.6470

Intrathecal EBV-specific antibody synthesis was defined using the submitted binary variables EBNA1-CAI ≥1.5 and EBV-CA-CAI ≥1.5. Values are n/N (%), where N is the number of patients with an available CAI measurement within the

corresponding serum Q4 stratum. Odds ratios (ORs) compare Q4 with <Q4 serum IgG levels and are shown with 95% confidence intervals. Two-sided p-values are from Fisher's exact tests because of small event counts. The four p-values were adjusted within each analysis panel (overall cohort, female patients, male patients) using the Benjamini–Hochberg false discovery rate procedure. For the single male comparison containing a zero event cell, a 0.5 continuity correction was applied for estimation of the OR and its confidence interval; the Fisher's exact p-value was calculated without correction. Sex-stratified analyses were exploratory.

Abbreviations: CAI, CSF/serum antigen-specific antibody index; CI, confidence interval; EBNA1, Epstein–Barr virus nuclear antigen 1; EBV-CA, Epstein–Barr virus capsid antigen; FDR, false discovery rate; OR, odds ratio; Q4, highest quartile.

Supplementary Figures

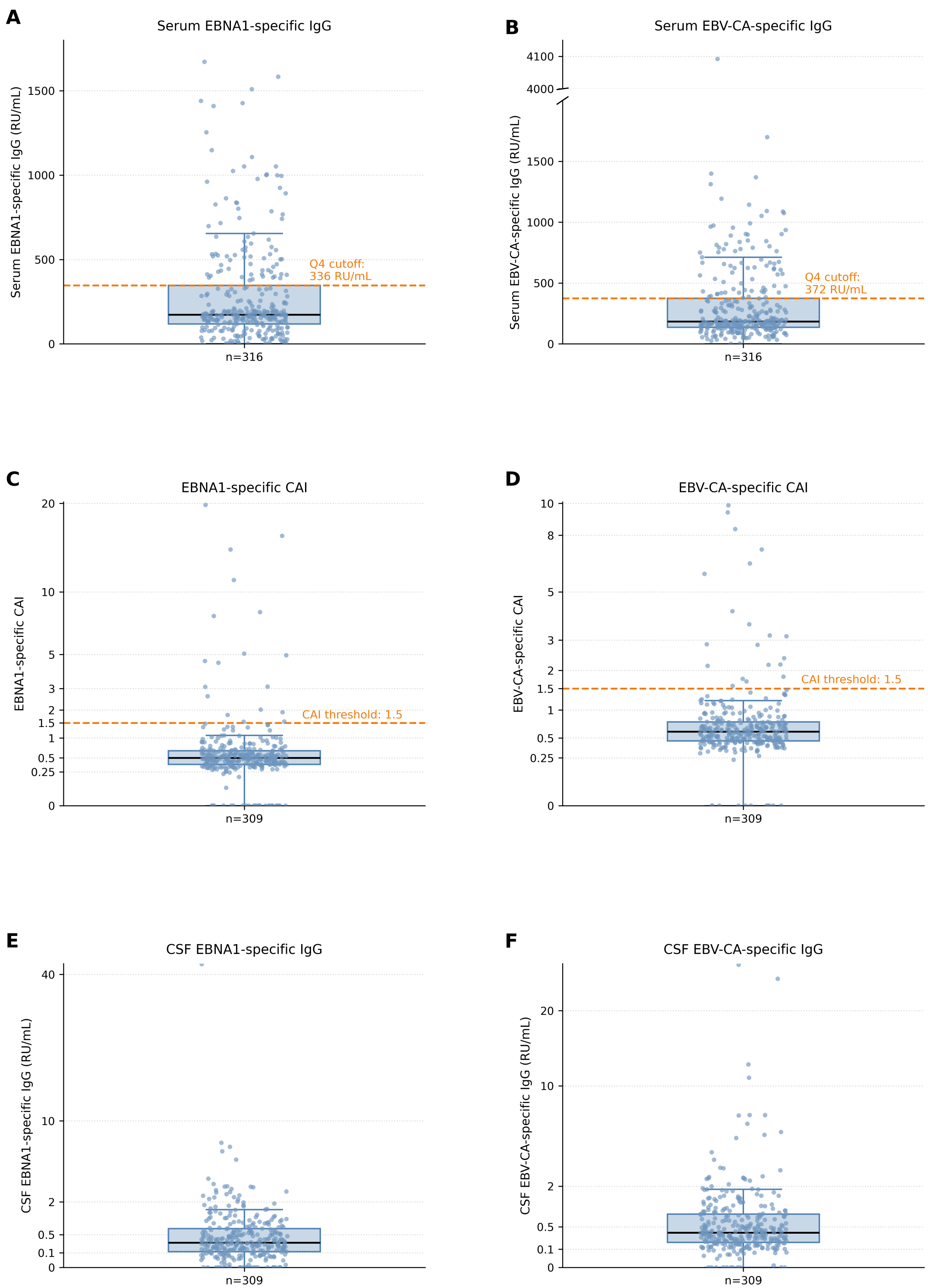

Supplementary Figure 1. Distribution of serum and CSF EBV-specific IgG measures in the study cohort. Box-and-whisker plots with overlaid individual patient-level observations show serum

EBNA1-specific IgG concentrations (A), serum EBV capsid antigen (EBV-CA)-specific IgG concentrations (B), EBNA1-specific CSF/serum antibody indices (CAI) (C), EBV-CA-specific CSF/serum antibody indices (CAI) (D), CSF EBNA1-specific IgG concentrations (E), and CSF EBV-CA-specific IgG concentrations (F). Boxes indicate the interquartile range, horizontal black lines indicate medians, and whiskers extend to the most extreme observations within 1.5× the interquartile range; individual observations are overlaid to visualize the full distribution. In panels A and B, orange dashed lines indicate the Q4 cutoffs used in the main analyses (336 RU/mL for serum EBNA1-specific IgG and 372 RU/mL for serum EBV-CA-specific IgG). In panels C and D, orange dashed lines indicate the CAI threshold of 1.5 used to define intrathecal EBV-specific antibody production. Panels C–F use nonlinear y-axis scaling to improve visualization of the dense lower range while retaining all observations. CSF EBV-specific IgG concentrations in panels E and F were derived from the corresponding serum concentrations and antigen-specific CAI values according to the Reiber antibody-index relationship, using the total IgG quotient or the corresponding Reiber upper reference limit, as appropriate. The number of observations included in each panel is indicated below the respective plot.

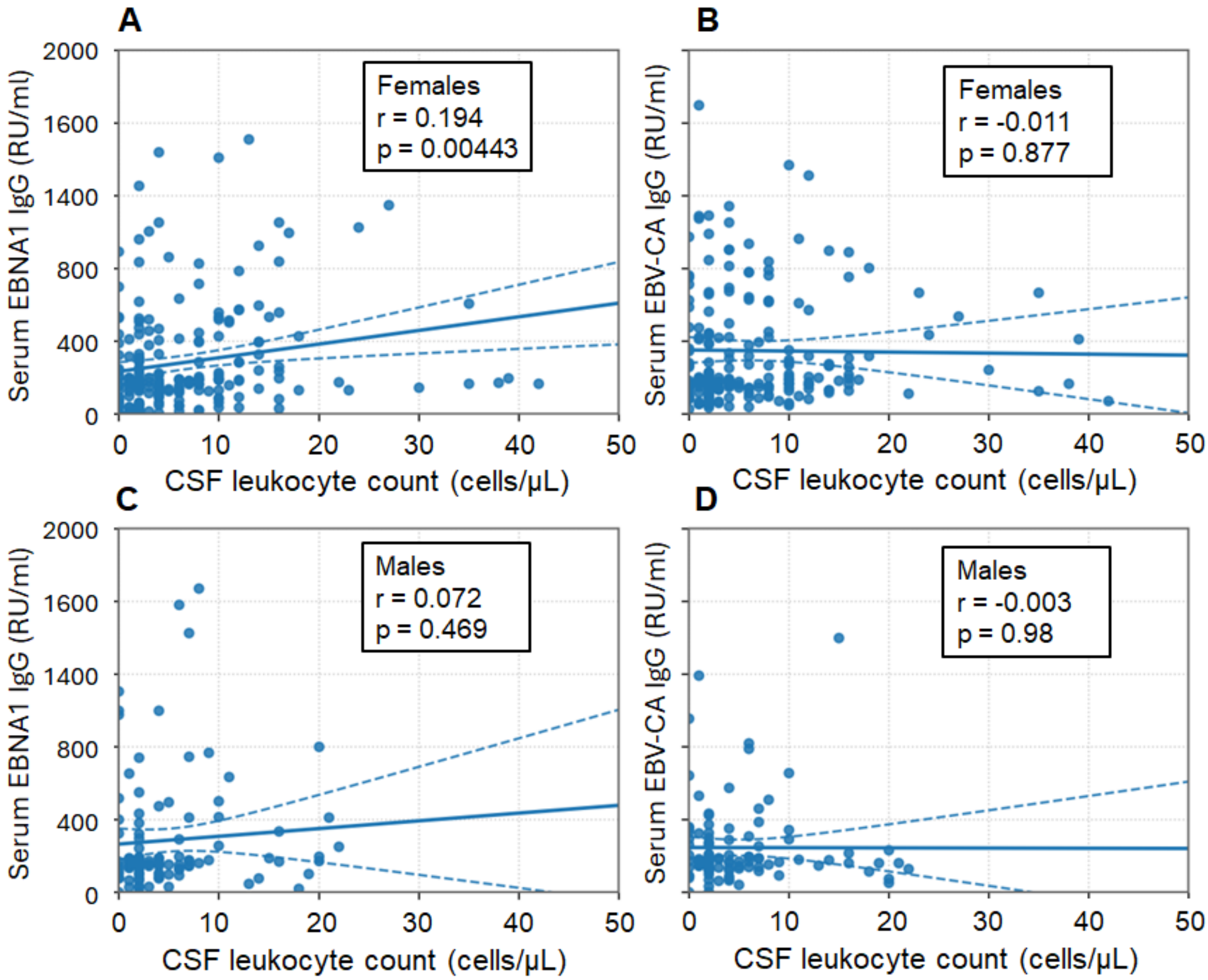

Supplementary Figure 2. Correlations between serum EBV-specific IgG levels and CSF leukocyte count among female and male MS patients. Scatter plot of serum EBV-specific IgG (RU/mL; x-axis) versus CSF leukocyte count (cells/µL; y-axis) among female and male patients. The solid line indicates the fitted linear regression, with the shaded band showing the 95% confidence interval. The correlation coefficient (Pearson's r) and corresponding p value are displayed in the panel. Each dot represents one MS patient. A) Significant correlation between serum EBNA1-specific IgG levels and CSF leukocyte count among female MS patients. B) No significant correlation between serum EBV-CA-specific IgG levels and CSF leukocyte count among female MS patients. C) No significant correlation between serum EBNA1-specific IgG levels and CSF leukocyte count among male MS patients. D) No significant correlation between serum EBV-CA-specific IgG levels and CSF leukocyte count among female and male MS patients.

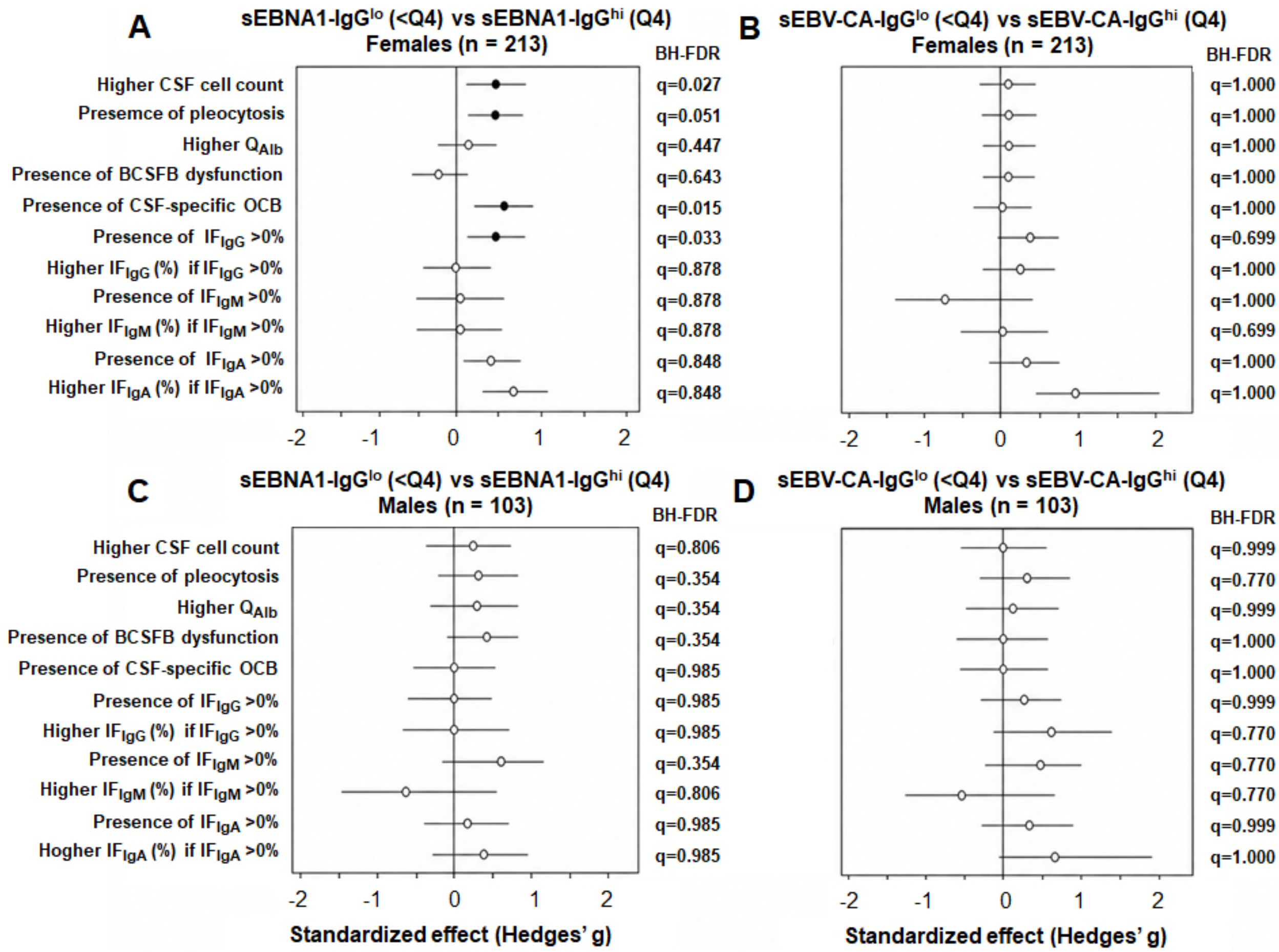

Supplementary Figure 3. Association maps comparing MS patients with versus without high serum EBV-specific IgG levels (Q4 versus <Q4 levels) stratified by sex. Standardized differences in basic CSF parameters among (A and B) female and (C and D) male MS patients with and without high serum EBNA1-specific IgG levels (sEBNA1-IgG^{lo} vs sEBNA1-IgG^{hi}; A and C) or high serum EBV-CA-specific IgG levels (sEBV-CA-IgG^{lo} vs sEBV-CA-IgG^{hi}; B and D) (Q4 versus <Q4). Effect sizes are reported as Hedges' g with 95% confidence intervals. Benjamini–Hochberg false discovery rate (BH-FDR) correction was applied within each stratum and exposure panel across the displayed outcomes, and q-values are shown in the right-side column. Filled points denote q<0.05 and open points denote q≥0.05. Outcome definitions and ordering match the original Figure 2 to facilitate visual comparison across strata and include (i) CSF parameters of cellular inflammation, i.e. CSF leukocyte count, and presence of pleocytosis (CSF leukocyte count >4 cells/µL); (ii) parameters of blood-CSF barrier dysfunction (BCSFB), i.e. Q_{Alb} (CSF/serum albumin quotient) and presence of BCSFB; and (iii) parameters of general intrathecal humoral immune activation, i.e. presence of CSF-specific OCB (oligoclonal bands type 2 or 3), presence of IF_{IgG} >0% (intrathecal fraction of IgG >0%, i.e. intrathecal production), IF_{IgG} or IF_{IgG} (%) if IF_{IgG} >0%), as well intrathecal fraction of total IgA and total IgM.

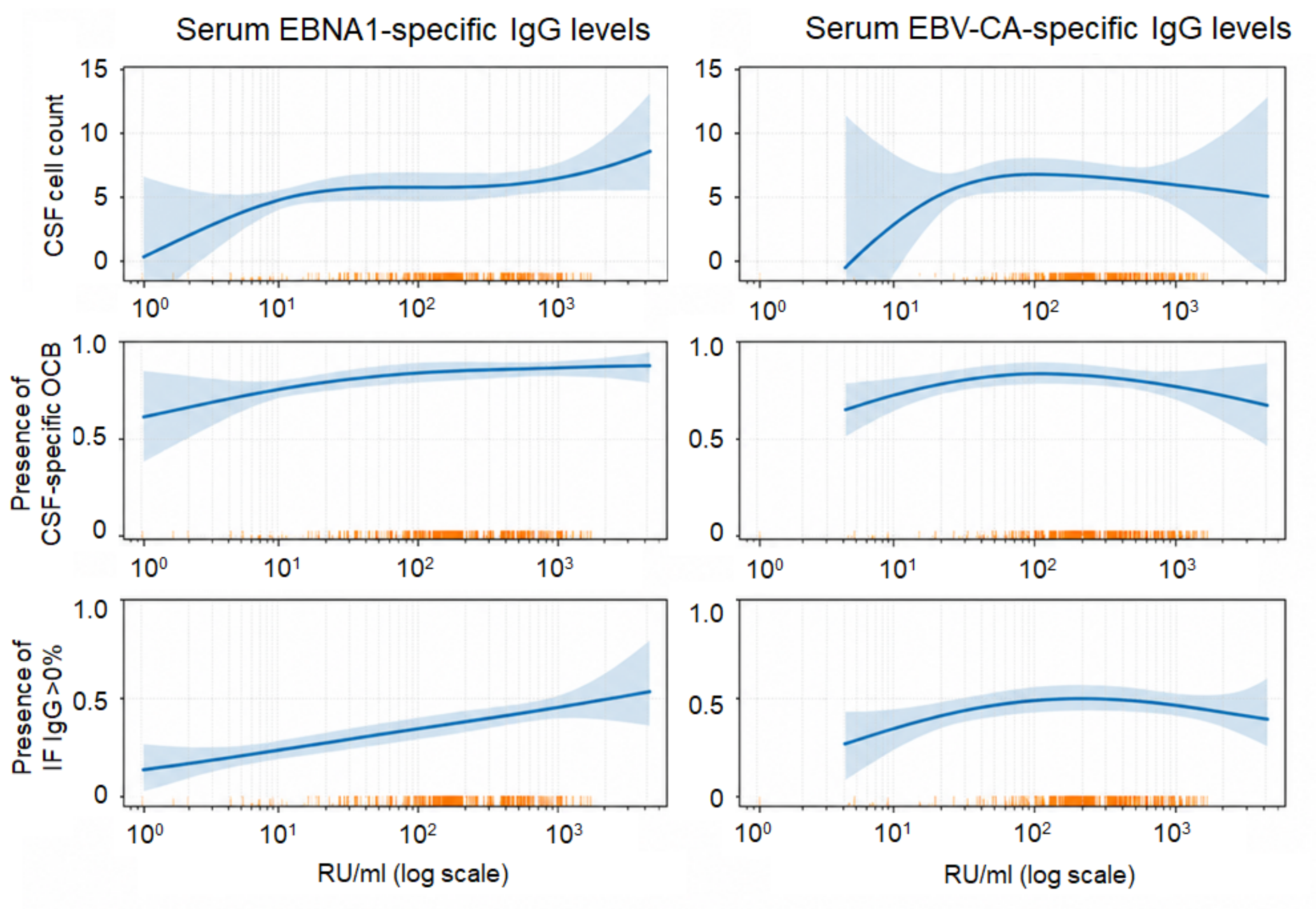

Supplementary Figure 4. Restricted cubic spline dose–response models for serum EBNA1-specific and EBV-CA-specific IgG concentrations. Restricted cubic spline analyses of serum EBNA1-specific IgG levels (left column) and serum EBV-CA-specific IgG levels (right column) were performed to assess potential non-linear exposure–response relationships. The x-axis displays antibody concentrations (RU/mL) on a log10 scale. Outcomes and plotting conventions match Figure 3: (i) CSF leukocyte count (cells/µL; log1p-modelled and back-transformed), (ii) presence of CSF-restricted OCB (oligoclonal bands types 2/3; binary), and (iii) presence of IF_{IgG} >0% (intrathecal fraction of IgG >0%, i.e. intrathecal production of total IgG according to Reiber 1998; binary). Solid lines indicate the spline-based predicted associations and shaded bands indicate 95% confidence intervals. Rug marks in orange show the distribution of observed serum EBNA1-specific and EBV-CA-specific antibody concentration values. Wider confidence intervals at extreme antibody concentrations reflect sparse observations.

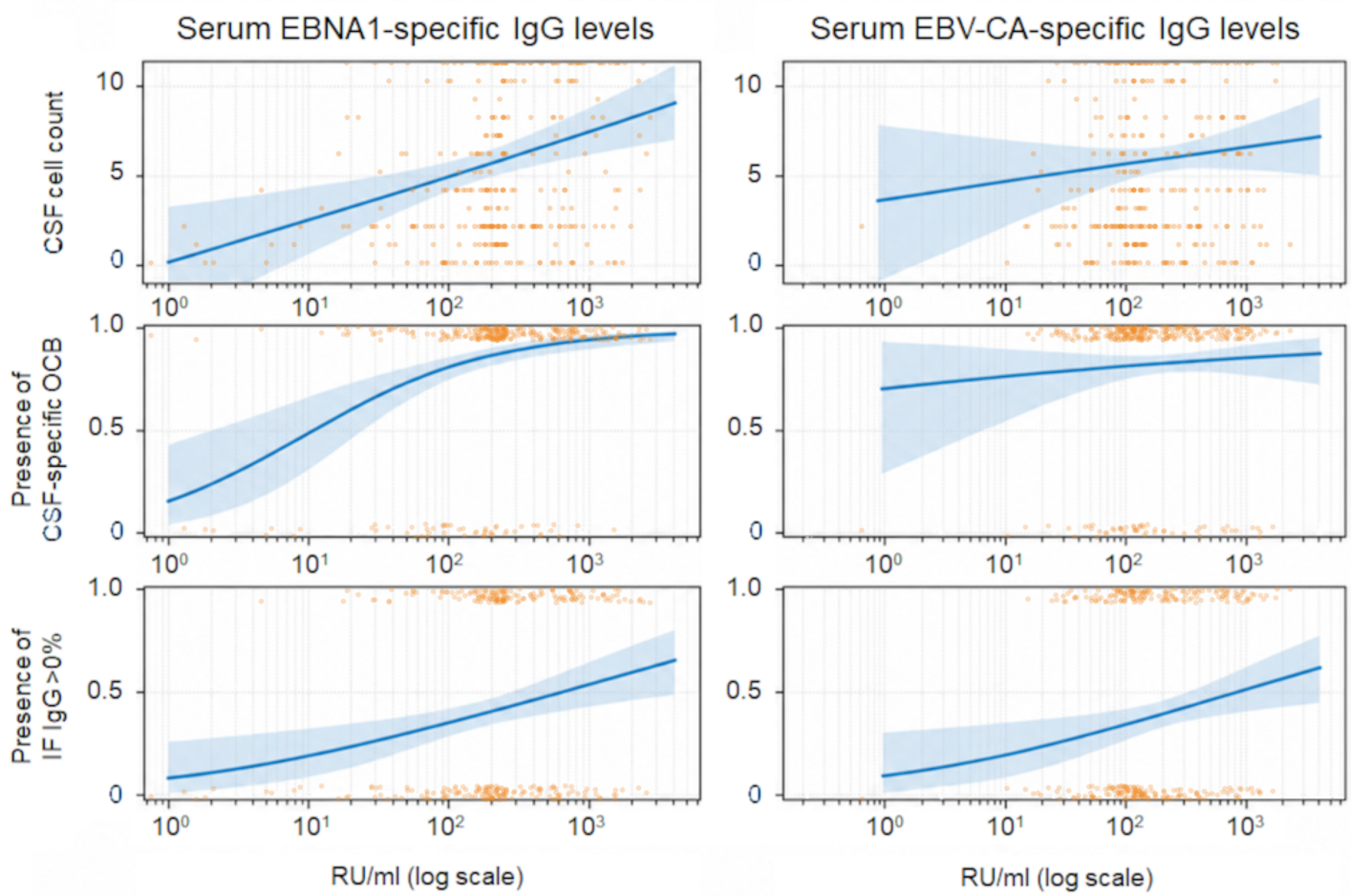

Supplementary Figure 5. Empirical observations overlaid on model-based dose–response relationships between serum EBV-specific IgG concentrations and selected CSF inflammatory parameters. Individual patient-level observations are overlaid on the model-based dose–response relationships shown in Figure 3 to allow direct visual comparison of the empirical data with the fitted predictions. The left column shows serum EBNA1-specific IgG levels and the right column serum EBV-CA-specific IgG levels, modelled as continuous exposures. Serum antibody concentrations are displayed in RU/mL on a logarithmic x-axis. Outcomes are CSF leukocyte count (top row; cells/µL), presence of CSF-specific oligoclonal bands (OCB; middle row), and presence of intrathecal total IgG synthesis according to Reiber (IF_{IgG} >0%; bottom row). Solid blue lines indicate the model-predicted relationships and blue shaded bands the corresponding 95% confidence intervals. Orange points represent the individual observed outcome values. For the binary outcomes, the fitted curves represent predicted probabilities, whereas the observed values are binary patient-level outcomes displayed with slight vertical jitter to reduce overplotting; jittering is used for visualization only and does not affect model estimation.
